# Structural Variations in the Central Heterocyclic Scaffold of Tripartite 2,6-Difluorobenzamides: Influence on Their Antibacterial Activity against MDR *Staphylococcus aureus*

**DOI:** 10.3390/molecules27196619

**Published:** 2022-10-05

**Authors:** Thibaut Barbier, Cédric Badiou, Floriane Davy, Yves Queneau, Oana Dumitrescu, Gérard Lina, Laurent Soulère

**Affiliations:** 1 Univ Lyon, INSA Lyon, Université Claude Bernard Lyon 1, CNRS, CPE-Lyon, ICBMS, UMR 5246, Institut de Chimie et de Biochimie Moléculaires et Supramoléculaires, Bâtiment Lederer, 1 Rue Victor Grignard, 69622 Villeurbanne, France; 2 Team STAPATH, CIRI, Centre International de Recherche en Infectiologie, INSERM, U1111, Université Claude Bernard Lyon 1, CNRS, UMR5308, ENS de Lyon, 69007 Lyon, France; 3 Hospices Civils de Lyon, Hôpital de la Croix Rousse-Centre de Biologie Nord, Institut des Agents Infectieux, Laboratoire de Bactériologie, Grande Rue de la Croix Rousse, 69004 Lyon, France

**Keywords:** azoles, antibacterial, *Staphylococcus aureus*, benzamide

## Abstract

Five series of heterocyclic tripartite 2,6-difluorobenzamides, namely 1,2,3-triazoles, 1,2,4- and 1,3,4-oxadiazoles, analogs of reported model anti-staphylococcal compounds, were prepared. The purpose was to investigate the influence of the nature of the heterocyclic central scaffold on the biological activity against three strains of *S. aureus,* including two drug-resistant ones. Among the 15 compounds of the new collection, a 3-(4-*tert*-butylphenyl)-1,2,4-oxadiazole linked via a methylene group with a 2,6-difluorobenzamide moiety (**II.c**) exhibited a minimal inhibitory concentration between 0.5 and 1 µg/mL according to the strain. Subsequent studies on **II.c** demonstrated no human cytotoxicity, while targeting the bacterial divisome.

## 1. Introduction

**Figure 1:**
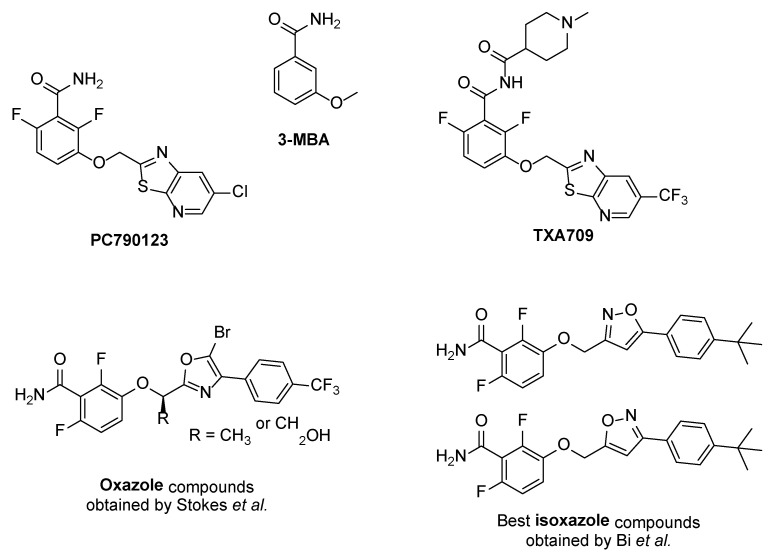
Structure of several reported FtsZ inhibitors as antibacterial agents.

Antimicrobial resistance is a worldwide health problem concerning many pathogens including *Staphylococcus aureus* [1,2]. Compounds with a 2,6-difluorobenzamide moiety are described as interesting antibacterial agents targeting the protein FtsZ of the divisome (Figure 1). Among this class of compounds, 3-methoxybenzamide was first discovered [3] and was the starting point for a great number of derivatives, such as the pyridinothiazole PC190723 [4] and the prodrug TXA709 [5]. Several 2,6-difluorobenzamide derivatives bearing an oxazole [6], an isoxazole [7,8], an oxadiazol-2-one [9], and an oxadiazole ring [10] were also described as interesting FtsZ inhibitors (Figure 1). For the latter, only activity ranges were described, rendering difficult accurate SAR studies.

**Figure 2:**
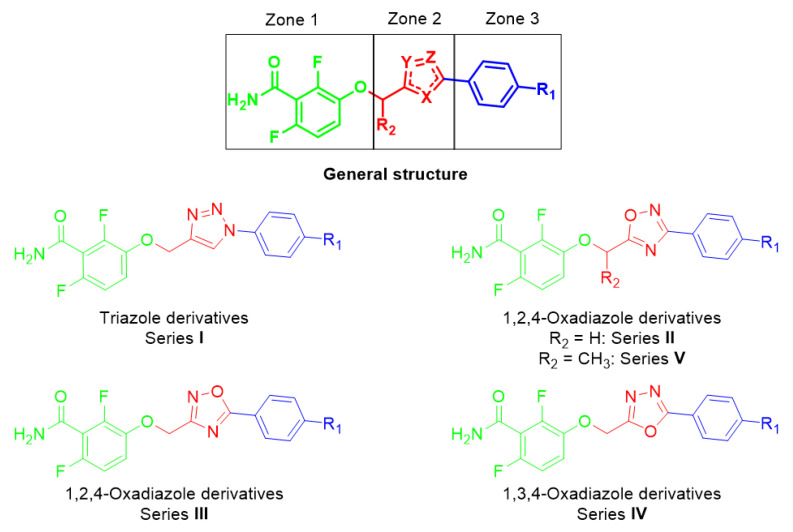
General structure of the compounds in this study.

The general structure of the oxazole or isoxazole derivatives can be divided in three zone: the 2,6-difluorobenzamide moiety, a central five-member ring linked to a methylene group (substituted or not), and the substituted phenyl group (Figure 2). Among reported compounds, there has not been any systematic study of the nature of the central heterocyclic scaffold, with or without oxygen atom, or with the oxygen atom located at all possible positions of the ring. To this aim, we prepared a collection of analogs including 1,2,3-triazole derivatives, two series of 1,2,4-oxadiazoles, and one series of 1,3,4-oxadiazoles (Figure 2) which were evaluated against three *S. aureus* strains.

## 2. Results and Discussion

### 2.1. Chemistry

Based on the literature, Zone 3 was established as a phenyl group, by means of reference, and as a 4-trifluoromethyl-phenyl group or 4-*tert*-butylphenyl group, considering their beneficial effect on the biological activity against *S. aureus* for other tripartite compounds reported in previous studies [6,7].

**Scheme 1:**
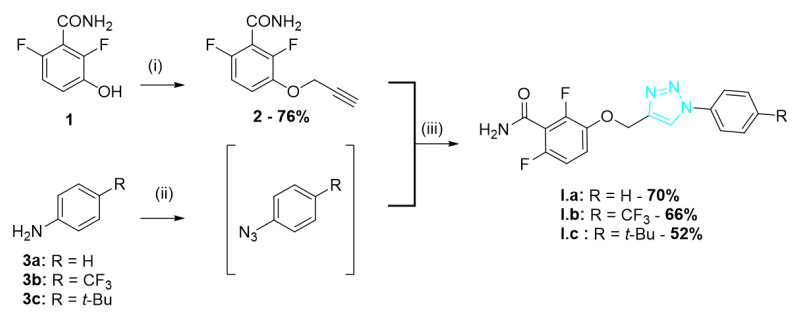
Synthesis of series **I** triazole derivatives. Reagents and conditions: (**i**) propargyl bromide, K_2_CO_3_, acetone, r.t., 24 h; (**ii**) *t*-BuONO, TMSN_3_, ACN, 0 °C then r.t., 7 h; and (**iii**) CuI, DIPEA, r.t., 16 h.

The synthetic route for the series **I** of triazole derivatives is shown in Scheme 1. Benzamide **1** synthetized as previously described [9] was alkylated with propargyl bromide to afford alkyne **2**. Various anilines **3** were converted into the corresponding azides using *tert*-butylnitrite and trimethylsilyl azide [11] and were then cyclized with **2** through a click reaction using copper (I) iodide to give the triazoles **I.a**–**c**.

**Scheme 2:**
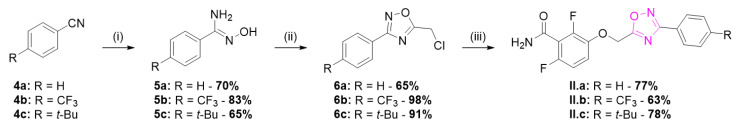
Synthesis of series **II** 1,2,4-oxadiazole derivatives. Reagents and conditions: (**i**) HONH_2_·HCl, Et_3_N, EtOH, 80 °C, 2 h; (**ii**) chloroacetyl chloride, Et_3_N, toluene, 0 °C, 15 min then reflux, 4 h; and (**iii**) **1**, K_2_CO_3_, DMF, 35 °C, 3 h.

The synthetic route for the series **II** of 1,2,4-oxadiazoles is described in Scheme 2. The series **II** was obtained with various benzonitriles **4** converted to corresponding *N*-hydroxybenzimidamides **5 [12]** and acylated with chloroacetyl chloride and cyclized to give compounds **6** [13]. Alkylation of benzamide **1** afforded the oxadiazoles **II.a**–**c** [14].

**Scheme 3:**
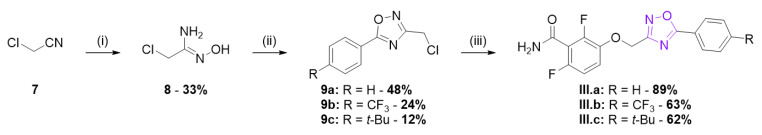
Synthesis of series **III** 1,2,4-oxadiazole derivatives. Reagents and conditions: (**i**) HONH_2_·HCl, Na_2_CO_3_, H_2_O, 0 °C, 2 h then r.t., 2 h; (**ii**) RPhCOCl, Et_3_N, toluene, 0 °C, 15 min then reflux, 4 h; and (**iii**) **1**, K_2_CO_3_, DMF, 35 °C, 3 h.

The other 1,2,4-oxadiazoles from series **III** were obtained as described in Scheme 3. Chloroacetonitrile was converted in 2-chloro-*N*-hydroxyacetimidamide **8** which was then acylated by various benzoyl chlorides and cyclized to afford compounds **9**. Subsequent alkylation of benzamide **1** afforded the oxadiazoles **III.a**–**c**.

**Scheme 4:**
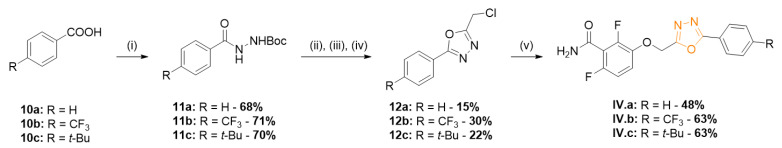
Synthesis of series **IV** 1,3,4-oxadiazole derivatives. Reagents and conditions: (**i**) H_2_N-NHBoc, DCC, DMAP, DCM, 0 °C then r.t., 16 h; (**ii**) TFA, DCM, r.t., 16 h; (**iii**) chloroacetyl chloride, Et_3_N, toluene, 0 °C, 15 min; and (**iv**) POCl_3_, 110 °C, 3 h; (**v**) **1**, K_2_CO_3_, DMF, 35 °C, 3 h.

The synthetic route for series **IV** is described in Scheme 4. Various benzoic acids **10** and *tert*-butyl carbazate was coupled using DCC as coupling agent. The Boc protecting group was cleaved using TFA, the free hydrazine was acylated by chloroacetyl chloride, and the compound was cyclized in presence of POCl_3_ [15] to give compounds **12**. Alkylation of benzamide **1** afforded the oxadiazoles **IV.a**–**c**.

### 2.2. Biological Evaluation

The series **I**, **II**, **III**, and **IV** were tested for their antimicrobial activity on three strains of *S. aureus*, including a reference (ATCC 29213) and two clinically isolated strains respectively resistant to methicillin (SF8300) [16] and daptomycin (ST20171643) [17], with PC190723 as control. The purpose of this experiment was to establish the minimum inhibitory concentration (MIC), which is the minimum concentration of compound needed to prevent the growth of a standardized bacterial inoculum, for the triazole and oxadiazole series. The results are presented in Table 1.

The triazole and the 1,3,4-oxadiazole compounds were found deprived of any antimicrobial activity against *S. aureus* at a concentration up to 256 µg/mL. Compounds from series **II**, with a 1,2,4-oxadiazole ring, had a better anti-staphylococcal activity than the oxadiazoles regioisomers of series **III** considering the same substitution of the phenyl ring. The 4-*tert*-butylphenyl substituent led to an improved activity for all series compared with other substituents, leading to the identification of compound **II.c** which had an MIC value between 0.5 and 1 µg/mL according to the staphylococcal strain. Interestingly, the activity in each series **I**–**IV** was correlated with the calculated LogP with the substituents *t*-Bu > CF_3_ > H. Additionally, between the series **I**–**IV** with the same phenyl substitution, an increased LogP correlated with an improved activity. The 1,2,4-oxadiazole derivative **II.c** was thus found to be particularly active with a lower MIC value compared with the corresponding isoxazole derivative [7].

### 2.3. Optimization of Series II

**Scheme 5:**
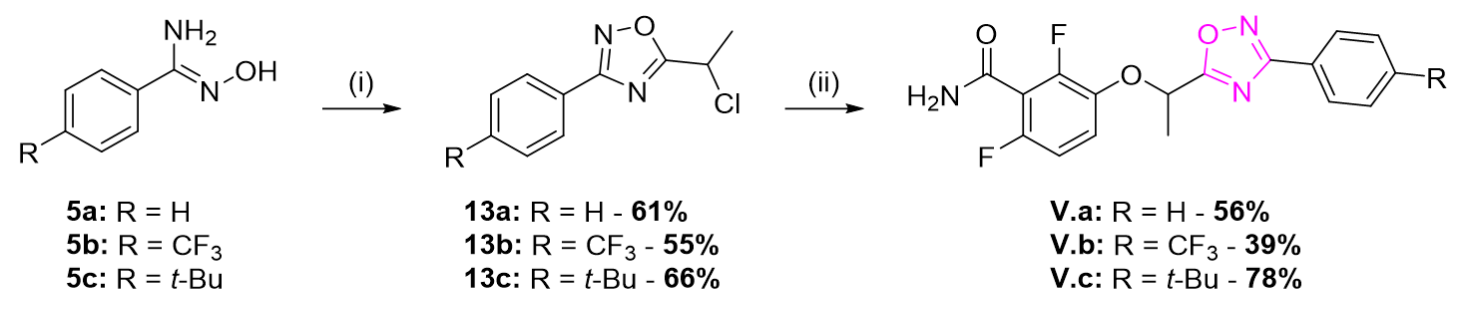
Synthesis of series **V** methyl-substituted 1,2,4-oxadiazole derivatives. Reagents and conditions: (**i**) chloropropionyl chloride, Et_3_N, toluene, 0 °C, 15 min then reflux, 4 h; (**ii**) **1**, K_2_CO_3_, DMF, 35 °C, 3 h.

The promising MIC values obtained for **II.c** led to a structural optimization of this compound in order to improve its anti-staphylococcal activity. As described in the literature [6], the addition of a hydrophobic methyl group on the methylene group between the phenol and the five members heteroaromatic ring could improve the biological activity. As a consequence, the synthetic route for series **V** was investigated and is shown in Scheme 5. The *N*-hydroxybenzimidamides **5** were acylated with 2-chloropropionyl chloride and cyclized to give compounds **13**. Alkylation of benzamide **1** afforded the oxadiazoles **V.a-c**. Enantiopure synthesis was first attempted, but partial racemization occurred. The study of racemic compounds was, therefore, preferred, by using racemic chloropropionyl chloride during their synthesis.

The MIC values for series **V** are shown in Table 2. Interestingly, the additional hydrophobic methyl group significantly increases the antibacterial ability for the less active compounds (phenyl and trifluoromethylphenyl derivatives), reaching 1 µg/mL for **V.b**, comparable to the MIC values of the most active **II.c**.

### 2.4. Mechanism of Action

**Figure 3:**
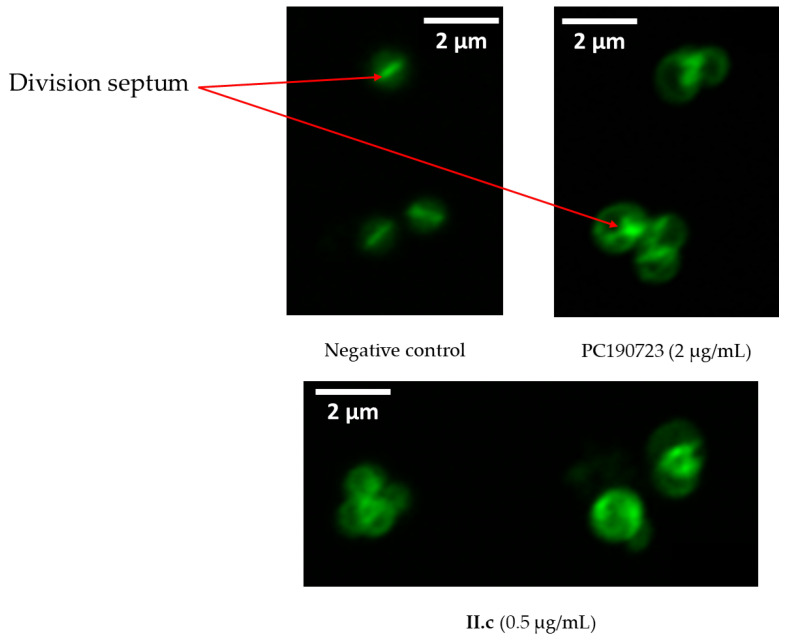
Fluorescence microscope images of ATCC 29213 bacteria stained with vancomycin-bodipy conjugate in presence or absence of antibacterial agent.

Filamentous temperature-sensitive mutant Z (FtsZ) is an essential protein for bacterial cytokinesis, a homologue to mammalian tubulin. During division, GTP-bound FtsZ polymerizes into protofilaments to form the Z-ring, a mid-cell scaffold, allowing for the recruitment and assembly of other division proteins [18,19]. Therefore, FtsZ inhibition is detrimental for the bacteria and is leading to cell death, and this protein is considered to be a valuable pharmacological target [20,21,22,23,24,25]. Since 2,6-difluorobenzamide derivatives are regularly described as FtsZ inhibitors and exert an alteration of the morphology of the bacteria by inducing aberrant localization of division septum, the action of **II.c** on the bacterial divisome was studied by cell wall staining. Bacteria (strain ATCC 29213) were incubated with compound **II.c** at the minimal inhibitory concentration for 4 h before fluorescent staining of the wall with a vancomycin-bodipy conjugate [26]. PC190723 was used as a positive control. As presented in Figure 3, dividing control bacteria are small (1 µm of diameter) with a centered division septum. On the contrary, bacteria incubated with **II.c** show the same morphology as bacteria incubated with PC190723, including larger overall size (approximately 1.5 µm of diameter) and wrongly localized division septum, indicating bacterial divisome targeting. Identical results were obtained with SF8300 (MRSA) *S. aureus* (see Figure S1, Supplementary Information). These results, added to structural similarities between **II.c** and described compounds, strongly suggest FtsZ inhibition.

### 2.5. Molecular Docking

A docking study of **II.c** was performed on the crystal structure of *S. aureus* FtsZ within the allosteric binding site (PDB code 6KVP [27]). The side chain of 13 amino acids of this allosteric pocket (namely Met98, Val129, Ile197, Leu200, Val203, Asn208, Leu209, Val214, Met218, Ile228, Asn263, Val297, and Thr309) were set as flexible in order to best mimic potential movements of the pocket which was found to be dynamic [28].

**Figure 4:**
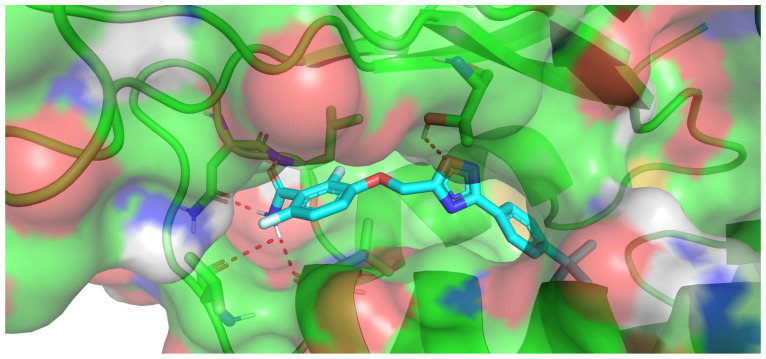
Docking results within the allosteric binding site of FtsZ obtained for compound **II.c**.

The three parts of the molecule show multiple interactions with the protein (Figure 4): the benzamide moiety forms an extensive hydrogen bond network with the protein, the *tert*-butylphenyl group is deeply inserted in the hydrophobic part of the pocket, generating van der Waals interactions, and the oxadiazole ring is able to form an H-bond with Thr309. Therefore, the position of the oxygen atom plays a central role for the activity of compounds of series **II** by increasing their lipophilicity and by possibly interacting with Thr309 via H-bond. The importance of the presence of an oxygen atom has been also observed in benzodioxane derivatives [29,30]. Docking simulations of this type of compounds and of compound **II.c** led to a superimposition of the oxygen atoms (see Figure S2, Supplementary Information). Docking simulations of both enantiomers of compounds **V.b** and **V.c** were also performed and showed good superimpositions between the (R) derivatives and the co-crystallized inhibitor [6] (see Figure S3, Supplementary Information). These results are in agreement with the literature for substituted oxazole derivatives, showing better activity for (R) enantiomers.

### 2.6. Cytotoxicity Assay

**Figure 5:**
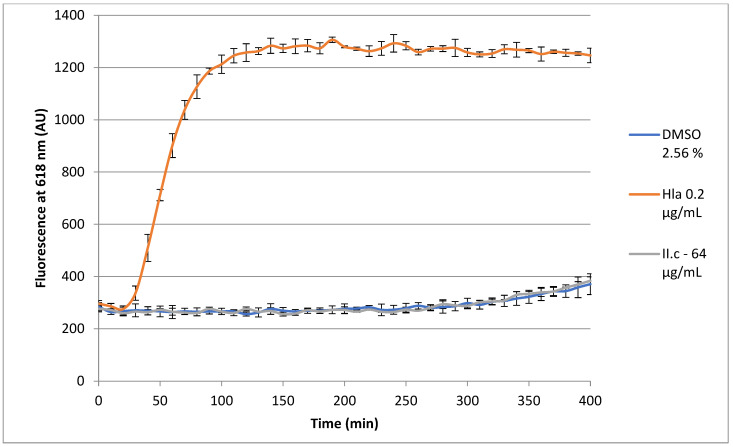
Effect of **II.c** on the growth of A549 cells.

Due to its interesting anti-staphylococcal activity, the potential mammalian cytotoxicity of **II.c** was examined. Therefore, a 7 h propidium iodide (PI)-based assay was used on adenocarcinomic human alveolar basal epithelial cells (A549), with recombinant *S. aureus* α-hemolysin as positive control. As depicted in Figure 5, a fluorescent maximum was reached within two hours in the presence of α-hemolysin. A small rise was observed for negative control and **II.c** after 6 h. The two curves are superimposed, showing an absence of cytotoxicity for **II.c** after 7 h at a concentration of 64 µg/mL (64 times the MIC).

## 3. Materials and Methods

All commercial materials were used as received without further purification. Flash chromatography was carried out using Macherey–Nagel Kieselgel 60 M silica. Analytical thin-layer chromatography was realized using aluminum-backed plates coated with Macherey–Nagel Kieselgel 60 XtraSIL G/UV254 and were visualized under UV light (at 254 nm or 365 nm) or stained using ninhydrin. Nuclear magnetic resonance (NMR) spectra were recorded on Bruker AV300, Bruker AV400, or Bruker AV500 spectrometers, operating at 300 MHz, 400 MHz, and 500 MHz, respectively, for the proton (^1^H) NMR and at 75 MHz, 100 MHz, and 125 MHz, respectively, for the carbon (^13^C) NMR. Chemical shifts were reported in parts per million (ppm) on a scale relative to residual solvent signals. Multiplicities are abbreviated as: s, singlet; d, doublet; t, triplet; q, quadruplet; dd, doublet of doublets; dt, doublet of triplets; td, triplet of doublets; ddd, doublet of doublet of doublets; and m, multiplet. Coupling constants were measured in Hertz (Hz). Copies of NMR spectra of final products are available in Supplementary Materials. High-resolution mass spectra (HRMS) and low-resolution mass spectra were obtained by the Centre Commun de Spectrométrie de Masse (CCSM), University of Lyon 1, Lyon, France. LogP calculations were performed using Molinspiration Cheminformatics free web services, https://www.molinspiration.com (accessed on 6 December 2021), Slovensky Grob, Slovakia.

### 3.1. Chemistry

#### 3.1.1. Series I

**Procedure for 2,6-difluoro-3-(prop-2-yn-1-yloxy)benzamide**

To a solution of 2,6-difluoro-3-hydroxybenzamide (173 mg, 1.0 mmol, 1.0 eq.) in DMF (2 mL) were added K_2_CO_3_ (276 mg, 2.0 mmol, 2.0 eq.), NaI (15 mg, 0.10 mmol, 0.10 eq.), and propargyl bromide (80% wt in toluene, 145 µL, 1.3 mmol, 1.3 eq.). The solution was stirred at 35 °C for 3 h. The mixture was then diluted with water, extracted with EtOAc, dried over Na_2_SO_4_, and concentrated under vacuum. The residue was then purified by chromatography (eluent: pentane/EtOAc 1:1).

**2,6-Difluoro-3-(prop-2-yn-1-yloxy)benzamide (2)**

White powder (161 mg, 76% yield). ^1^H NMR (300 MHz, DMSO-*d*_6_) δ 8.16 (s, 1H), 7.87 (s, 1H), 7.29 (td, *J* = 9.1, 5.3 Hz, 1H), 7.12 (td, *J* = 9.1, 1.9 Hz, 1H), 4.91 (d, *J* = 2.4 Hz, 2H), 3.65 (t, *J* = 2.4 Hz, 1H); ^13^C NMR (125 MHz, DMSO-*d*_6_) δ 161.2, 152.3 (dd, *J* = 241.8, 7.0 Hz), 148.2 (dd, *J* = 248.5, 8.2 Hz), 141.5 (dd, *J* = 10.8, 3.1 Hz), 116.7 (dd, *J* = 24.9, 20.6 Hz), 116.3 (dd, *J* = 9.6, 2.2 Hz), 110.9 (dd, *J* = 23.0, 3.9 Hz), 79.1, 78.6, 57.0.

**Procedure for 2,6-difluoro-3-((1-(phenyl)-1*H*-1,2,3-triazol-4-yl)methoxy)benzamides**

To a solution of 4-substituted aniline (0.24 mmol, 1.0 eq.) in ACN (1 mL) at 0 °C were added dropwise *tert*-butyl nitrite (42 µL, 0.36 mmol, 1.5 eq.) and trimethylsilyl azide (38 µL, 0.28 mmol, 1.2 eq.). The solution was then stirred at room temperature for 3 h. 2,6-Difluoro-3-(prop-2-yn-1-yloxy) benzamide (50 mg, 0.24 mmol, 1.0 eq.), CuI (2 mg, 0.012 mmol, 0.05 eq.), and three drops of DIPEA were then added to the solution. The mixture was stirred at room temperature for 16 h. The solvent was then evaporated, and the residue was purified by chromatography (eluent pentane/EtOAc 1:1).

**2,6-Difluoro-3-((1-phenyl-1*H*-1,2,3-triazol-4-yl)methoxy)benzamide (I.a)**

White powder (69 mg, 70% yield). ^1^H NMR (300 MHz, Acetone-*d*_6_) δ 8.71 (s, 1H), 7.97–7.87 (m, 2H), 7.69–7.56 (m, 2H), 7.55–7.48 (m, 1H), 7.47–7.38 (m, 2H), 7.18 (s, 1H), 6.99 (td, *J* = 8.9, 1.9 Hz, 1H), 5.37 (s, 2H); ^13^C NMR (125 MHz, DMSO-*d*_6_) δ 161.4, 152.1 (dd, *J* = 241.5, 6.7 Hz), 148.1 (dd, *J* = 248.6, 8.3 Hz), 143.2, 142.5 (dd, *J* = 10.9, 3.2 Hz), 136.6, 123.0, 128.9, 123.3, 120.3, 116.7 (dd, *J* = 24.7, 20.6 Hz), 116.1 (d, *J* = 9.2 Hz), 111.0 (dd, *J* = 22.9, 3.9 Hz), 62.6; HRMS (ESI) m/z: calcd. for C_16_H_13_F_2_N_4_O_2_ [M+H]^+^ 331.1001, found 331.0996.

**2,6-Difluoro-3-((1-(4-(trifluoromethyl)phenyl)-1*H*-1,2,3-triazol-4-yl)methoxy)benzamide (I.b)**

White powder (63 mg, 66% yield). ^1^H NMR (400 MHz, Acetone-*d*_6_) δ 8.87 (s, 1H), 8.20 (d, *J* = 8.5 Hz, 2H), 7.97 (d, *J* = 8.5 Hz, 2H), 7.48 (s, 1H), 7.42 (td, *J* = 9.1, 5.2 Hz, 1H), 7.21 (s, 1H), 6.99 (td, *J* = 9.1, 2.1 Hz, 1H), 5.39 (s, 2H); ^13^C NMR (100 MHz, Acetone-*d*_6_) δ 162.2, 153.9 (dd, *J* = 242.8, 6.6 Hz), 150.0 (dd, *J* = 250.2, 8.1 Hz), 145.1, 143.8 (dd, *J* = 11.1, 3.6 Hz), 140.7, 130.7 (q, *J* = 32.5 Hz), 128.0 (q, *J* = 3.9 Hz), 125.3 (q, *J* = 271.7 Hz), 123.5, 121.6, 117.6 (dd, *J* = 9.5, 2.9 Hz), 117.8–117.1 (m), 111.6 (dd, *J* = 23.4, 4.3 Hz), 64.0; HRMS (ESI) m/z: calcd. for C_17_H_11_F_5_N_4_NaO_2_ [M+Na]^+^ 421.0694, found 421.068.

**3-((1-(4-*Tert*-butylphenyl)-1*H*-1,2,3-triazol-4-yl)methoxy)-2,6-difluorobenzamide (I.c)**

Yellow powder (48 mg, 52% yield). ^1^H NMR (500 MHz, Acetone-*d*_6_) δ 8.67 (s, 1H), 7.82 (d, *J* = 8.8 Hz, 2H), 7.64 (d, *J* = 8.8 Hz, 1H), 7.47 (s, 1H), 7.42 (td, *J* = 9.1, 5.2 Hz, 1H), 7.20 (s, 1H), 6.99 (td, *J* = 9.1, 2.1 Hz, 1H), 5.36 (s, 2H), 1.37 (s, 9H); ^13^C NMR (125 MHz, Acetone-*d*_6_) δ 162.2, 153.9 (dd, *J* = 242.7, 6.5 Hz), 152.7, 150.0 (dd, *J* = 250.1, 8.2 Hz), 144.5, 143.9 (dd, *J* = 11.2, 3.4 Hz), 135.7, 127.5, 123.2, 120.9, 117.5 (dd, *J* = 9.5, 2.8 Hz), 117.6–117.3 (m), 111.5 (dd, *J* = 23.2, 4.1 Hz), 64.0, 35.3, 31.5; HRMS (ESI) m/z: calcd. for C_20_H_21_F_2_N_4_O_2_ [M+H]^+^ 387.1627, found 387.1644.

#### 3.1.2. Series II

**General procedure for *N*-hydroxybenzimidamides**

To a solution of 4-substituted benzonitrile (2.0 mmol, 1.0 eq.) in EtOH (4 mL) were added triethylamine (418 µL, 3.0 mmol, 1.5 eq.) and hydroxylamine hydrochloride (208 mg, 3.0 mmol, 1.5 eq.). The solution was stirred at reflux for 2 h, and the solvent was then evaporated. The residue was dissolved in water, extracted with EtOAc, dried over Na_2_SO_4_, and concentrated under vacuum. The residue was then purified by chromatography (eluent pentane/EtOAc 2:1).

***N*-hydroxybenzimidamide (5a)**

White powder (192 mg, 70% yield). ^1^H NMR (300 MHz, Acetone-*d*_6_) δ 8.94 (s, 1H), 7.77–7.68 (m, 2H), 7.41–7.31 (m, 3H), 5.47 (s, 2H).

[Spectrum in accordance with Lin et al. 2014 [31]].

***N*-hydroxy-4-(trifluoromethyl)benzimidamide (5b)**

White powder (338 mg, 83% yield). ^1^H NMR (500 MHz, DMSO-*d*_6_) δ 9.93 (s, 1H), 7.90 (d, *J* = 8.2 Hz, 2H), 7.74 (d, *J* = 8.2 Hz, 2H), 5.99 (s, 2H).

[Spectrum in accordance with Lin et al. 2014 [31]].

**4-*Tert*-butyl-*N*-hydroxybenzimidamide (5c)**

White powder (503 mg, 65%). ^1^H NMR (500 MHz, Acetone-*d*_6_) δ 9.08 (s, 1H), 7.66 (dt, *J* = 8.7, 1.9 Hz, 2H), 7.42 (dt, *J* = 8.7, 1.9 Hz, 2H), 5.47 (s, 2H), 1.31 (s, 9H).

[Spectrum in accordance with Xu et al. 2018 [32]].

**General procedure for 5-chloromethyl-3-phenyl-1,2,4-oxadiazoles**

To a solution of 4-substituted *N*-hydroxybenzimidamide (0.49 mmol, 1.03 eq.) and triethylamine (66 µL, 0.47 mmol, 1.0 eq.) in toluene (1 mL) at 0 °C was added dropwise chloroacetyl chloride (38 µL, 0.47 mmol, 1.0 eq.) diluted in toluene (1 mL). The solution was stirred at 0 °C for 15 min, and then at 110 °C for 4 h. The mixture was then cooled to room temperature, extracted with toluene, washed with water and brine, dried over Na_2_SO_4_, and concentrated under vacuum to give the desired product which was used without further purification.

**5-Chloromethyl-3-phenyl-1,2,4-oxadiazole (6a)**

Translucent oil (93 mg, 65% yield). ^1^H NMR (300 MHz, Chloroform-*d*) δ 8.13–8.02 (m, 2H), 7.62–7.41 (m, 3H), 4.75 (s, 2H).

[Spectrum in accordance with Cai et al. 2015 [33]].

**5-Chloromethyl-3-(4-(trifluoromethyl)phenyl)-1,2,4-oxadiazole (6b)**

Translucent oil (121 mg, 98% yield). ^1^H NMR (300 MHz, Chloroform-*d*) δ 8.22 (dt, *J* = 8.1, 0.8 Hz, 2H), 7.77 (dt, *J* = 8.1, 0.8 Hz, 2H), 4.77 (s, 2H).

[Spectrum in accordance with Cai et al. 2015 [33]].

**3-(4-*Tert*-butylphenyl)-5-chloromethyl-1,2,4-oxadiazole (6c)**

Translucent oil (117 mg, 91% yield). ^1^H NMR (500 MHz, Chloroform-*d*) δ 8.01 (dt, *J* = 8.6, 2.0 Hz, 2H), 7.51 (dt, *J* = 8.6, 2.0 Hz, 2H), 4.74 (s, 2H), 1.36 (s, 9H); ^13^C NMR (125 MHz, Chloroform-*d*) δ 174.3, 168.9, 155.2, 127.4, 126.0, 123.4, 35.1, 33.5, 31.3.

**General procedure for 2,6-difluoro-3-((3-phenyl-1,2,4-oxadiazol-5-yl)methoxy)benzamides**

To a solution of 2,6-difluoro-3-hydroxybenzamide (35 mg, 0.20 mmol, 1.0 eq.) in DMF (1 mL) were added K_2_CO_3_ (56 mg, 0.40 mmol, 2.0 eq.) and 3-substituted-5-chloromethyl-1,2,4-oxadiazole (0.20 mmol, 1.0 eq.). The solution was stirred at 35 °C for 3 h. The solution was extracted with EtOAc, washed with water and brine, dried over Na_2_SO_4_, and concentrated under vacuum. The residue was then purified by chromatography (eluent: pentane/EtOAc 1:1).

**2,6-Difluoro-3-((3-phenyl-1,2,4-oxadiazol-5-yl)methoxy)benzamide (II.a)**

Orange powder (66 mg, 77% yield). ^1^H NMR (300 MHz, Acetone-*d*_6_) δ 8.14–7.99 (m, 2H), 7.63–7.53 (m, 3H), 7.51 (s, 1H), 7.42 (td, *J* = 9.2, 5.1 Hz, 1H), 7.21 (s, 1H), 7.03 (ddd, *J* = 9.2, 8.6, 2.1 Hz, 1H), 5.65 (s, 2H); ^13^C NMR (125 MHz, Acetone-*d*_6_) δ 176.0, 169.3, 161.8, 154.6 (dd, *J* = 244.2, 6.6 Hz), 150.2 (dd, *J* = 250.8, 8.3 Hz), 143.3 (dd, *J* = 11.3, 3.4 Hz), 132.5, 130.0, 128.1, 127.3, 118.1 (dd, *J* = 9.5, 2.2 Hz), 117.8 (dd, *J* = 24.3, 20.1 Hz), 111.9 (dd, *J* = 23.6, 4.1 Hz), 63.7; HRMS (ESI) m/z: calcd. for C_16_H_12_F_2_N_3_O_3_ [M+H]^+^ 332.0841, found 332.0837.

**2,6-Difluoro-3-((3-(4-(trifluoromethyl)phenyl)-1,2,4-oxadiazol-5-yl)methoxy)benzamide (II.b)**

White powder (43 mg, 63% yield). ^1^H NMR (500 MHz, Acetone-*d*_6_) δ 8.29 (d, *J* = 7.8 Hz, 2H), 7.93 (d, *J* = 7.8 Hz, 2H), 7.52 (s, 1H), 7.43 (td, *J* = 9.0, 5.0 Hz, 1H), 7.25 (s, 1H), 7.03 (td, *J* = 9.0, 2.1 Hz, 1H), 5.68 (s, 2H); ^13^C NMR (125 MHz, Acetone-*d*_6_) δ 176.6, 168.3, 161.9, 154.7 (dd, *J* = 244.2, 6.7 Hz), 150.2 (dd, *J* = 251.0, 8.3 Hz), 143.2 (dd, *J* = 11.4, 3.4 Hz), 133.4 (q, *J* = 32.5 Hz), 131.1, 128.9, 127.0 (q, *J* = 3.8 Hz), 124.9 (q, *J* = 271.7 Hz), 118.2 (dd, *J* = 9.6, 2.3 Hz), 117.7 (dd, *J* = 24.5, 20.0 Hz), 111.9 (dd, *J* = 23.6, 4.1 Hz), 63.7; HRMS (ESI) m/z: calcd. for C_17_H_11_F_5_N_3_O_3_ [M+H]^+^ 400.0715, found 400.0716.

**3-((3-(4-*Tert*-butylphenyl)-1,2,4-oxadiazol-5-yl)methoxy)-2,6-difluorobenzamide (II.c)**

White powder (60 mg, 78% yield). ^1^H NMR (300 MHz, Acetone-*d*_6_) δ 8.03–7.97 (m, 2H), 7.65–7.59 (m, 2H), 7.50 (s, 1H), 7.41 (td, *J* = 9.3, 5.1 Hz, 1H), 7.21 (s, 1H), 7.03 (ddd, *J* = 9.2, 8.6, 2.1 Hz, 1H), 5.63 (s, 2H), 1.36 (s, 9H); ^13^C NMR (100 MHz, Acetone-*d_6_*) δ 175.8, 169.2, 162.0, 155.8, 154.2 (dd, *J* = 244.2, 6.9 Hz), 150.2 (dd, *J* = 250.9, 8.3 Hz), 143.3 (dd, *J* = 11.1, 3.6 Hz), 128.0, 126.9, 124.5, 118.1 (dd, *J* = 9.6, 2.2 Hz), 117.7 (d, *J* = 20.4 Hz), 111.9 (dd, *J* = 23.7, 4.1 Hz), 63.7, 35.5, 31.4; HRMS (ESI) m/z: calcd. for C_20_H_20_F_2_N_3_O_3_ [M+H]^+^ 388.1467, found 388.1473.

#### 3.1.3. Series III

**Procedure for 2-chloro-*N*-hydroxyacetimidamide**

To a solution of hydroxylamine hydrochloride (1.390 g, 20.0 mmol, 1.0 eq.) and Na_2_CO_3_ (1.060 g, 10 mmol, 0.5 eq.) in water (6 mL) at 0 °C was added dropwise chloroacetonitrile (1.266 mL, 20.0 mmol, 1.0 eq.) over 2 h. The mixture was then stirred for two additional hours. The mixture was then extracted with EtOAc, dried over Na_2_SO_4_, and concentrated under vacuum. The residue was then purified by chromatography (eluent: pentane/EtOAc 3:2).

**2-Chloro-*N*-hydroxyacetimidamide (8)**

White powder (713 mg, 33% yield). ^1^H NMR (300 MHz, Chloroform-*d*) δ 6.29 (s, 1H), 4.78 (s, 2H), 4.05 (s, 2H).

[Spectrum in accordance with McLeod et al. 2004 [34]].

**General procedure for 3-chloromethyl-5-phenyl-1,2,4-oxadiazoles**

To a solution of 4-substituted benzoic acid (1.0 mmol, 1.0 eq.) in DCM (4 mL) at 0 °C were added two drops of DMF and dropwise oxalyl chloride (0.5 M, 2.4 mL, 1.2 mmol, 1.2 eq.). The solution was stirred at room temperature for 2 h, and the solvent was evaporated. The residue was dissolved in toluene (2 mL) and added dropwise to a solution of 2-chloro-*N*-hydroxyacetimidamide (112 mg, 1.03 mmol, 1.03 eq.) and triethylamine (139 µL, 1.0 mmol, 1.0 eq.) in toluene (2 mL) at 0 °C. The solution was stirred at 0 °C for 15 min and then at 110 °C for 4 h. The mixture was then cooled to room temperature, extracted with toluene, washed with water and brine, dried over Na_2_SO_4_, and concentrated under vacuum. The residue was then purified by chromatography (eluent: pentane/Et_2_O 95:5).

**3-Chloromethyl-5-(4-(trifluoromethyl)phenyl)-1,2,4-oxadiazole (9b)**

Translucent oil (64 mg, 24% yield). ^1^H NMR (300 MHz, Chloroform-*d*) δ 8.28 (dt, *J* = 8.0, 0.8 Hz, 2H), 7.81 (dt, *J* = 8.0, 0.8 Hz, 2H), 4.69 (s, 2H).

[Spectrum in accordance with Ye et al. 2019 [35]].

**5-(4-*Tert*-butylphenyl)-3-chloromethyl-1,2,4-oxadiazole (9c)**

Translucent oil (29 mg, 12% yield). ^1^H NMR (300 MHz, Acetone-*d*_6_) δ 8.09 (dt, *J* = 8.8, 2.1 Hz, 2H), 7.69 (dt, *J* = 8.8, 2.1 Hz, 2H), 4.84 (s, 2H), 1.37 (s, 9H); ^13^C NMR (125 MHz, Acetone-*d*_6_) δ 177.3, 169.0, 157.9, 128.7, 127.3, 121.9, 35.8, 35.5, 31.3.

**3-Chloromethyl-5-phenyl-1,2,4-oxadiazole (9a)**

The first step consisted of adding benzoyl chloride (340 µL, 2.0 mmol, 1.0 eq.) dropwise to a solution of 2-chloro-*N*-hydroxyacetimidamide (217 mg, 2.0 mmol, 1.0 eq.) and triethylamine (279 µL, 2.0 mmol, 1.0 eq.) in toluene (2 mL) at 0 °C. The mixture was stirred at 0 °C for 15 min and then at 110 °C for 4 h. The next steps were as described before.

Translucent oil (183 mg, 48% yield). ^1^H NMR (300 MHz, Chloroform-*d*) δ 8.20–8.11 (m, 2H), 7.67–7.48 (m, 3H), 4.68 (s, 2H).

[Spectrum in accordance with Ye et al. 2019 [35]].

**General procedure for 2,6-difluoro-3-((5-phenyl-1,2,4-oxadiazol-3-yl)methoxy)benzamides**

To a solution of 2,6-difluoro-3-hydroxybenzamide (28 mg, 0.16 mmol, 1.0 eq.) in DMF (1 mL) were added K_2_CO_3_ (44 mg, 0.32 mmol, 2.0 eq.) and 5-substituted-3-chloromethyl-1,2,4-oxadiazole (0.16 mmol, 1.0 eq.). The solution was stirred at 35 °C for 3 h. The solution was extracted with EtOAc, washed with water and brine, dried over Na_2_SO_4_, and concentrated under vacuum. The residue was then purified by chromatography (eluent: pentane/EtOAc 1:1).

**2,6-Difluoro-3-((5-phenyl-1,2,4-oxadiazol-3-yl)methoxy)benzamide (III.a)**

White powder (121 mg, 89% yield). ^1^H NMR (500 MHz, Acetone-*d*_6_) δ 8.21–8.14 (m, 2H), 7.73 (tt, *J* = 7.5, 1.4 Hz, 1H), 7.65 (tt, *J* = 7.5, 1.4 Hz, 2H), 7.48 (s, 1H), 7.41 (td, *J* = 9.0, 5.1 Hz, 1H), 7.18 (s, 1H), 7.02 (td, *J* = 9.0, 2.1 Hz, 1H), 5.42 (s, 2H); ^13^C NMR (125 MHz, Acetone-*d*_6_) δ 177.2, 168.2, 162.0, 154.4 (dd, *J* = 243.5, 6.5 Hz), 150.2 (dd, *J* = 250.7, 8.2 Hz), 143.6 (dd, *J* = 11.2, 3.4 Hz), 134.2, 130.4, 128.9, 124.7, 117.9 (dd, *J* = 9.6, 2.3 Hz), 117.7 (dd, *J* = 24.6, 20.2 Hz), 111.7 (dd, *J* = 23.4, 4.2 Hz), 63.7; HRMS (ESI) m/z: calcd. for C_16_H_12_F_2_N_3_O_3_ [M+H]^+^ 332.0841, found 332.0842.

**2,6-Difluoro-3-((5-(4-(trifluoromethyl)phenyl)-1,2,4-oxadiazol-3-yl)methoxy)benzamide (III.b)**

White powder (43 mg, 63% yield). ^1^H NMR (500 MHz, Acetone-*d*_6_) δ 8.39 (dt, *J* = 8.1, 0.8 Hz, 2H), 8.00 (dt, *J* = 8.1, 0.8 Hz, 2H), 7.50 (s, 1H), 7.41 (td, *J* = 9.1, 5.1 Hz, 1H), 7.23 (s, 1H), 7.02 (td, *J* = 9.1, 2.1 Hz, 1H), 5.46 (s, 2H); ^13^C NMR (125 MHz, Acetone-*d*_6_) δ 175.9, 168.4, 162.0, 154.4 (dd, *J* = 243.6, 6.5 Hz), 150.2 (dd, *J* = 250.7, 8.2 Hz), 143.5 (dd, *J* = 11.3, 3.6 Hz), 134.6 (q, *J* = 32.6 Hz), 129.7, 128.2, 127.3 (q, *J* = 3.8 Hz), 124.7 (q, *J* = 272.0 Hz), 118.0 (dd, *J* = 9.7, 2.4 Hz), 117.6 (dd, *J* = 24.5, 20.0 Hz), 111.7 (dd, *J* = 23.5, 4.2 Hz), 63.7; HRMS (ESI) m/z: calcd. for C_17_H_11_F_5_N_3_O_3_ [M+H]^+^ 400.0715, found 400.0709.

**3-((5-(4-*Tert*-butylphenyl)-1,2,4-oxadiazol-3-yl)methoxy)-2,6-difluorobenzamide (III.c)**

White powder (28 mg, 62%). ^1^H NMR (500 MHz, Acetone-*d*_6_) δ 8.09 (dt, *J* = 8.7, 2.0 Hz, 2H), 7.69 (dt, *J* = 8.7, 2.0 Hz, 2H), 7.48 (s, 1H), 7.40 (td, *J* = 9.3, 5.1 Hz, 1H), 7.20 (s, 1H), 7.01 (ddd, *J* = 9.2, 8.7, 2.0 Hz, 1H), 5.41 (s, 2H), 1.37 (s, 9H); ^13^C NMR (125 MHz, Acetone-*d*_6_) δ 177.2, 168.1, 162.0, 157.8, 154.3 (dd, *J* = 243.5, 6.5 Hz), 150.2 (dd, *J* = 250.7, 8.2 Hz), 143.6 (dd, *J* = 11.3, 3.4 Hz), 128.7, 127.3, 122.0, 117.9 (dd, *J* = 9.5, 2.5 Hz), 117.6 (dd, *J* = 24.5, 20.0 Hz), 111.7 (dd, *J* = 23.4, 4.2 Hz), 63.8, 35.8, 31.3; HRMS (ESI) m/z: calcd. for C_20_H_20_F_2_N_3_O_3_ [M+H]^+^ 388.1467, found 388.1473.

#### 3.1.4. Series IV

**General procedure for *tert*-butyl 2-benzoylhydrazine-1-carboxylates**

To a solution of 4-substituted benzoic acid (1.1 mmol, 1.1 eq.) and *tert*-butyl carbazate (132 mg, 1.0 mmol, 1.0 eq.) in DCM (4 mL) at 0 °C were added gradually DMAP (16 mg, 0.13 mmol, 0.13 eq.) and DCC (248 mg, 1.2 mmol, 1.2 eq.). The solution was stirred at room temperature for 16 h. The mixture was then filtered on Celite^®^ and concentrated under vacuum. The residue was then purified by chromatography (eluent: pentane/EtOAc 5:1).

***Tert*-butyl 2-(4-(trifluoromethyl)benzoyl)hydrazine-1-carboxylate (11b)**

White powder (216 mg, 71% yield). ^1^H NMR (300 MHz, Chloroform-*d*) δ 8.54 (s, 1H), 7.89 (d, *J* = 8.1 Hz, 2H), 7.65 (d, *J* = 8.1 Hz, 2H), 6.76 (s, 1H), 1.50 (s, 9H); ^13^C NMR (100 MHz, Chloroform-*d*) δ 165.7, 156.7, 134.6, 133.8 (q, *J* = 32.9 Hz), 127.9, 125.6 (q, *J* = 3.7 Hz), 123.6 (q, *J* = 272.5 Hz), 82.6, 28.2.

***Tert*-butyl 2-(4-*tert*-butylbenzoyl)hydrazine-1-carboxylate (11c)**

White powder (205 mg, 70% yield). ^1^H NMR (300 MHz, Acetone-*d*_6_) δ 9.35 (s, 1H), 7.92 (s, 1H), 7.86 (d, *J* = 8.6 Hz, 2H), 7.53 (dt, *J* = 8.6, 2.0 Hz, 2H), 1.44 (s, 9H), 1.34 (s, 9H); ^13^C NMR (100 MHz, Acetone-*d*_6_) δ 166.4, 155.8, 155.0, 130.3, 127.3, 125.3, 79.5, 34.6, 30.5, 27.6.

***Tert*-butyl 2-benzoylhydrazine-1-carboxylate (11a)**

The first step consisted of adding benzoyl chloride (255 µL, 2.2 mmol, 1.1 eq.) dropwise to a solution of *tert*-butyl carbazate (264 mg, 2.0 mmol, 1.0 eq.) and triethylamine (613 µL, 4.4 mmol, 2.2 eq.) in DCM (3 mL) at 0 °C. The mixture was stirred at room temperature for 6 h, and then washed with water, extracted with DCM, dried over Na_2_SO_4_, and concentrated under vacuum. The purification was as described before.

White powder (321 mg, 68% yield). ^1^H NMR (300 MHz, Acetone-*d*_6_) δ 9.49 (s, 1H), 8.00 (s, 1H), 7.99–7.83 (m, 2H), 7.65–7.40 (m, 3H), 1.44 (s, 9H); ^13^C NMR (125 MHz, Acetone-*d*_6_) δ 167.4, 156.6, 133.9, 132.5, 129.3, 128.2, 80.4, 28.4.

**General procedure for 2-chloromethyl-5-phenyl-1,3,4-oxadiazoles**

To a solution of 4-substituted *tert*-butyl 2-benzoylhydrazine-1-carboxylate (0.33 mmol, 1.0 eq.) in DCM (2 mL) was added trifluoroacetic acid (2.013 mL, 26.3 mmol, 80 eq.). The solution was stirred at room temperature for 16 h, and the TFA was then co-evaporated with toluene. The residue was then dissolved in toluene (2 mL) at 0 °C, then triethylamine (101 µL, 0.72 mmol, 2.2 eq.) and dropwise chloroacetyl chloride (26 µL, 0.33 mmol, 1.0 eq.) were added. The solution was stirred at 0 °C for 15 min. POCl_3_ (30 µL, 0.33 mmol, 1.0 eq.) was then added and the solution was stirred at 110 °C for 4 h. The mixture was then cooled to room temperature, extracted with toluene, washed with water and brine, dried over Na_2_SO_4_, and concentrated under vacuum. The residue was then purified by chromatography (eluent: pentane/Et_2_O 4:1).

**2-Chloromethyl-5-phenyl-1,3,4-oxadiazole (12a)**

Translucent oil (24 mg, 15%). ^1^H NMR (300 MHz, Acetone-*d*_6_) δ 8.14–8.01 (m, 2H), 7.68–7.54 (m, 3H), 5.04 (s, 2H).

[Spectrum in accordance with Sharma et al. 2019 [36]].

**2-Chloromethyl-5-(4-(trifluoromethyl)phenyl)-1,3,4-oxadiazole (12b)**

Yellow oil (26 mg, 30% yield). ^1^H NMR (300 MHz, Chloroform-*d*) δ 8.21 (dt, *J* = 8.1, 0.8 Hz, 2H), 7.80 (dt, *J* = 8.1, 0.8 Hz, 2H), 4.81 (s, 2H).

[Spectrum in accordance with Sharma et al. 2019 [36]].

**2-(4-*Tert*-butylphenyl)-5-chloromethyl-1,3,4-oxadiazole (12c)**

Orange oil (28 mg, 22% yield). ^1^H NMR (300 MHz, Acetone-*d*_6_) δ 7.99 (d, *J* = 8.4 Hz, 2H), 7.66 (d, *J* = 8.4 Hz, 2H), 5.03 (s, 2H), 1.36 (s, 9H).

[Spectrum in accordance with Natero et al. 2004 [37]].

**General procedure for 2,6-difluoro-3-((5-phenyl-1,3,4-oxadiazol-2-yl)methoxy)benzamides**

To a solution of 2,6-difluoro-3-hydroxybenzamide (25 mg, 0.15 mmol, 1.3 eq.) in DMF (1 mL) were added K_2_CO_3_ (31 mg, 0.22 mmol, 2.0 eq.) and 5-substituted-2-chloromethyl-1,3,4-oxadiazole (0.11 mmol, 1.0 eq.). The solution was stirred at 35 °C for 3 h. The solution was extracted with EtOAc, washed with water and brine, dried over Na_2_SO_4_, and concentrated under vacuum. The residue was then purified by chromatography (eluent: pentane/EtOAc 1:1).

**2,6-Difluoro-3-((5-phenyl-1,3,4-oxadiazol-2-yl)methoxy)benzamide (IV.a)**

White powder (19 mg, 48% yield). ^1^H NMR (400 MHz, Acetone-*d*_6_) δ 8.10–8.04 (m, 2H), 7.68–7.58 (m, 3H), 7.50 (s, 1H), 7.46 (td, *J* = 9.1, 5.1 Hz, 1H), 7.19 (s, 1H), 7.04 (td, *J* = 9.1, 2.1 Hz, 1H), 5.56 (s, 2H); ^13^C NMR (100 MHz, Acetone-*d*_6_) δ 166.4, 163.1, 161.9, 155.1 (d, *J* = 203.1 Hz), 150.3 (d, *J* = 251.0 Hz), 143.3 (dd, *J* = 11.6, 3.8 Hz), 133.0, 130.2, 127.7, 124.6, 118.5 (d, *J* = 10.5 Hz), 118.1–117.4 (m), 111.9 (dd, *J* = 23.4, 4.4 Hz), 62.8; HRMS (ESI) m/z: calcd. for C_16_H_12_F_2_N_3_O_3_ [M+H]^+^ 332.0841, found 332.0840.

**2,6-Difluoro-3-((5-(4-(trifluoromethyl)phenyl)-1,3,4-oxadiazol-2-yl)methoxy)benzamide (IV.b)**

White powder (21 mg, 63% yield). ^1^H NMR (500 MHz, Acetone-*d*_6_) δ 8.30 (d, *J* = 8.0 Hz, 2H), 7.98 (d, *J* = 8.0 Hz, 2H), 7.51 (s, 1H), 7.46 (td, *J* = 9.1, 5.1 Hz, 1H), 7.25 (s, 1H), 7.04 (td, *J* = 9.1, 2.0 Hz, 1H), 5.59 (s, 2H); ^13^C NMR (125 MHz, Acetone-*d*_6_) δ 165.3, 163.8, 161.9, 154.7 (dd, *J* = 244.2, 6.7 Hz), 150.4 (dd, *J* = 250.7, 8.1 Hz), 143.2 (dd, *J* = 11.5, 3.4 Hz), 133.7 (q, *J* = 32.5 Hz), 128.5, 128.3, 127.2 (q, *J* = 3.8 Hz), 124.8 (q, *J* = 271.7 Hz), 118.6 (dd, *J* = 9.6, 2.0 Hz), 117.7 (dd, *J* = 24.3, 20.1 Hz), 111.9 (dd, *J* = 23.5, 4.3 Hz), 62.8; HRMS (ESI) m/z: calcd. for C_17_H_11_F_5_N_3_O_3_ [M+H]^+^ 400.0715, found 400.0723.

**3-((5-(4-*Tert*-butylphenyl)-1,3,4-oxadiazol-2-yl)methoxy)-2,6-difluorobenzamide (IV.c)**

White powder (27 mg, 63% yield). ^1^H NMR (500 MHz, Acetone-*d*_6_) δ 7.99 (dt, *J* = 8.7, 1.9 Hz, 2H), 7.66 (dt, *J* = 8.6, 1.9 Hz, 2H), 7.50 (s, 1H), 7.45 (td, *J* = 9.2, 5.1 Hz, 1H), 7.21 (s, 1H), 7.04 (ddd, *J* = 9.2, 8.6, 2.0 Hz, 1H), 5.54 (s, 2H), 1.36 (s, 9H); ^13^C NMR (125 MHz, Acetone-*d*_6_) δ 166.4, 162.8, 161.9, 156.5, 154.6 (dd, *J* = 244.1, 6.6 Hz), 150.4 (dd, *J* = 250.7, 8.2 Hz, 143.2 (dd, *J* = 11.4, 3.5 Hz), 127.6, 127.2, 121.8, 118.5 (dd, *J* = 9.6, 2.3 Hz), 117.7 (dd, *J* = 24.6, 20.1 Hz), 111.9 (dd, *J* = 23.6, 4.1 Hz), 62.7, 35.7, 31.3; HRMS (ESI) m/z: calcd. for C_20_H_20_F_2_N_3_O_3_ [M+H]^+^ 388.1467, found 388.1448.

#### 3.1.5. Series V

**General procedure for 5-(1-chloroethyl)-3-phenyl-1,2,4-oxadiazoles**

To a solution of 4-substituted *N*-hydroxybenzimidamide (0.40 mmol, 1.0 eq.) and triethylamine (67 µL, 0.48 mmol, 1.2 eq.) in toluene (3 mL) at 0 °C was added dropwise chloropropionyl chloride (47 µL, 0.48 mmol, 1.2 eq.) diluted in toluene (1 mL). The solution was stirred at 0 °C for 15 min, and then at 110 °C for 4 h. The mixture was then cooled to room temperature, extracted with toluene, washed with water and brine, dried over Na_2_SO_4_, and concentrated under vacuum. The residue was then purified by chromatography (eluent: pentane/Et_2_O 98:2).

**5-(1-Chloroethyl)-3-phenyl-1,2,4-oxadiazole (13a)**

Translucent oil (51 mg, 61% yield). ^1^H NMR (400 MHz, Chloroform-*d*) δ 8.13–8.06 (m, 2H), 7.55–7.45 (m, 3H), 5.23 (q, *J* = 7.0 Hz, 1H), 2.02 (d, *J* = 7.0 Hz, 3H); ^13^C NMR (100 MHz, Chloroform-*d*) δ 177.8, 168.8, 131.6, 129.0, 127.6, 126.4, 45.9, 22.7.

**5-(1-Chloroethyl)-3-(4-(trifluoromethyl)phenyl)-1,2,4-oxadiazole (13b)**

Translucent oil (61 mg, 55% yield). ^1^H NMR (400 MHz, Chloroform-*d*) δ 8.22 (dt, *J* = 8.0, 0.8 Hz, 2H), 7.75 (dt, *J* = 8.0, 0.8 Hz, 2H), 5.24 (q, *J* = 7.0 Hz, 1H), 2.03 (d, *J* = 7.0 Hz, 3H); ^13^C NMR (100 MHz, Chloroform-*d*) δ 178.4, 167.8, 133.3 (q, *J* = 32.7 Hz), 129.8 (d, *J* = 1.4 Hz), 128.0, 126.1 (q, *J* = 3.9 Hz), 122.5 (q, *J* = 272.2 Hz), 45.8, 22.6.

**3-(4-*Tert*-butylphenyl)-5-(1-chloroethyl)-1,2,4-oxadiazole (13c)**

Translucent oil (70 mg, 66% yield). ^1^H NMR (400 MHz, Chloroform-*d*) δ 8.02 (dt, *J* = 8.6, 2.0 Hz, 2H), 7.51 (dt, *J* = 8.6, 2.0 Hz, 2H), 5.23 (q, *J* = 7.0 Hz, 1H), 2.02 (d, *J* = 7.0 Hz, 3H), 1.36 (s, 9H); ^13^C NMR (100 MHz, Chloroform-*d*) δ 177.6, 168.7, 155.1, 127.4, 126.0, 123.6, 45.9, 35.1, 31.3, 22.7.

**General procedure for 2,6-difluoro-3-(1-(3-phenyl-1,2,4-oxadiazol-5-yl)ethoxy)benzamides**

To a solution of 2,6-difluoro-3-hydroxybenzamide (47 mg, 0.27 mmol, 1.1 eq.) in DMF (1 mL) were added K_2_CO_3_ (69 mg, 0.50 mmol, 2.0 eq.) and 3-substituted-5-(1-chloroethyl)-1,2,4-oxadiazole (0.25 mmol, 1.0 eq.). The solution was stirred at 35 °C for 3 h. The solution was extracted with EtOAc, washed with water and brine, dried over Na_2_SO_4_, and concentrated under vacuum. The residue was then purified by chromatography (eluent: pentane/EtOAc 2:1).

**2,6-Difluoro-3-(1-(3-phenyl-1,2,4-oxadiazol-5-yl)ethoxy)benzamide (V.a)**

White powder (34 mg, 56% yield). ^1^H NMR (400 MHz, Acetone-*d*_6_) δ 8.11–8.03 (m, 2H), 7.65–7.50 (m, 4H), 7.35 (td, *J* = 9.2, 5.2 Hz, 1H), 7.30 (s, 1H), 6.99 (td, *J* = 9.0, 2.1 Hz, 1H), 5.84 (q, *J* = 6.6 Hz, 1H), 1.90 (d, *J* = 6.7 Hz, 3H); ^13^C NMR (100 MHz, Acetone-*d*_6_) δ 179.0, 169.1, 162.0, 154.9 (dd, *J* = 245.0, 6.6 Hz), 150.9 (dd, *J* = 250.9, 8.6 Hz), 142.5 (dd, *J* = 11.7, 3.6 Hz), 132.4, 129.9, 128.1, 127.3, 120.2 (dd, *J* = 9.5, 2.2 Hz), 117.6 (dd, *J* = 24.4, 20.3 Hz), 112.0 (dd, *J* = 23.6, 4.0 Hz), 71.9, 19.6; HRMS (ESI) m/z: calcd. for C_17_H_14_F_2_N_3_O_3_ [M+H]^+^ 346.0998, found 346.0995.

**2,6-Difluoro-3-(1-(3-(4-(trifluoromethyl)phenyl)-1,2,4-oxadiazol-5-yl)ethoxy)benzamide (V.b)**

White powder (27 mg, 39% yield). ^1^H NMR (300 MHz, Acetone-*d*_6_) δ 8.29 (dt, *J* = 8.0, 0.8 Hz, 2H), 7.93 (dt, *J* = 8.0, 0.8 Hz, 2H), 7.50 (s, 1H), 7.37 (td, *J* = 9.2, 5.2 Hz, 1H), 7.21 (s, 1H), 6.99 (ddd, *J* = 9.2, 8.6, 2.0 Hz, 1H), 5.88 (q, *J* = 6.6 Hz, 1H), 1.91 (d, *J* = 6.6 Hz, 3H); ^13^C NMR (100 MHz, Acetone-*d*_6_) δ 179.6, 168.2, 162.0, 155.0 (dd, *J* = 245.0, 6.6 Hz), 151.0 (dd, *J* = 250.9, 8.6 Hz), 142.5 (dd, *J* = 11.7, 3.6 Hz), 133.4 (q, *J* = 32.3 Hz), 131.1, 128.9, 127.0 (q, *J* = 3.8 Hz), 123.6 (q, *J* = 271.4 Hz), 120.4 (dd, *J* = 9.9, 2.5 Hz), 117.7 (dd, *J* = 24.3, 20.4 Hz), 112.0 (dd, *J* = 23.5, 4.3 Hz), 72.0, 19.5; HRMS (ESI) m/z: calcd. for C_18_H_13_F_5_N_3_O_3_ [M+H]^+^ 414.0872, found 414.0864.

**3-(1-(3-(4-*Tert*-butylphenyl)-1,2,4-oxadiazol-5-yl)ethoxy)-2,6-difluorobenzamide (V.c)**

White powder (67 mg, 66% yield). ^1^H NMR (400 MHz, Acetone-*d*_6_) δ 7.99 (dt, *J* = 8.6, 2.0 Hz, 2H), 7.61 (dt, *J* = 8.6, 2.0 Hz, 2H), 7.50 (s, 1H), 7.34 (td, *J* = 9.1, 5.3 Hz, 1H), 7.22 (s, 1H), 6.99 (td, *J* = 9.1, 2.1 Hz, 1H), 5.83 (q, *J* = 6.6 Hz, 1H), 1.89 (d, *J* = 6.6 Hz, 3H), 1.36 (s, 9H); ^13^C NMR (100 MHz, Acetone-*d*_6_) δ 178.9, 169.0, 161.9, 155.8, 154.9 (dd, *J* = 244.4, 6.6 Hz), 151.0 (dd, *J* = 250.8, 8.3 Hz), 142.5 (dd, *J* = 11.1, 3.7 Hz), 128.0, 126.9, 124.6, 120.2 (dd, *J* = 9.9, 2.5 Hz), 117.7 (dd, *J* = 24.5, 20.2 Hz), 111.9 (dd, *J* = 23.9, 4.2 Hz), 71.9, 35.5, 31.4, 19.6; HRMS (ESI) m/z: calcd. for C_21_H_22_F_2_N_3_O_3_ [M+H]^+^ 402.1624, found 402.1616.

### 3.2. Biological Evaluation

#### 3.2.1. Minimum Inhibitory Concentration (MIC) Evaluation

MICs were evaluated in CaMHB (cation-adjusted Mueller–Hinton broth) by the method of microdilution in liquid medium which follows the CLSI recommendations (Clinical and Laboratory Standards Institute (CLSI), methods for dilution antimicrobial susceptibility tests for bacteria that grow aerobically; approved standard seventh edition. Clinical and Laboratory Standards Institute, Wayne, PA). Evaluated compounds were diluted in DMSO with a concentration of 5 mg/mL and then further diluted in CaMHB. The 0.5 MacFarland bacterial suspensions were made from colonies previously grown on blood agar plate (COS, Biomérieux) in a saline solution (0.45% NaCl); they were diluted in CaMHB (1/100) before addition into 96-well microplates. MICs were calculated in triplicate and determined after 18 h of incubation at 37 °C. The median values were reported.

#### 3.2.2. Divisome Inhibition Assay

Briefly, bacteria were grown for 18 h in BHI (Brain Heart Infusion) with 0.125 M of D-serine, to promote the incorporation of this amino acid into the cell wall. The culture was diluted to an OD_600_ of 0.05 into the same medium containing either **II.c** (0.5 µg/mL), PC190723 (2 µg/mL), or nothing (negative control) and grown in a 96-well plate with agitation at 37 °C for 4 h. Bacteria were then centrifuged and resuspended in the same volume of BHI with neither D-serine nor antibiotic. The bacteria were then incubated for 15 min at room temperature to allow the incorporation of D-alanine into the cell wall and then labeled with a mixture of equal amounts of vancomycin (Sigma) and a BODIPY FL conjugate of vancomycin (Van-FL, Molecular Probes) at the final concentration of 1 µg/mL, for 5 min at room temperature with agitation. Bacteria were then analyzed by fluorescence microscopy using a Zeiss LSM 880 Airyscan confocal microscope.

#### 3.2.3. Cytotoxicity Assay

Cytotoxicity was evaluated using a propidium iodide assay. Briefly, human lung adenocarcinomic A549 cells were plated on black flat 96-well plates (Greiner, Pleidelsheim, Germany) at a concentration of 0.5 × 10^6^ cells/mL in DMEM GlutaMAX (Gibco, Waltham, MA, USA) supplemented with 10% fetal bovine serum for 24 h in a humidified atmosphere at 37 °C and 5% CO_2_. The medium was then removed, and fresh medium with 2.5 µg/mL of propidium iodide and tested compounds (64 µg/mL of **II.c** and 0.2 µg/mL for recombinant Hla) was added. Plates were incubated for 7 h at 37 °C, and the cytotoxicity induced was evaluated by measuring propidium iodide (PI) incorporation into cells exposed to toxin or compounds using a fluorescence cell sorter (Spark R, TECAN, Zürich, Switzerland). Each concentration was made as a triplicate, and mean values are displayed with standard deviation.

### 3.3. Docking Studies

Compounds were drawn on Chem3D (PerkinElmer Informatics, Waltham, MA, USA), minimized with MM2 forcefield and saved as mol files. Docking was performed on FtsZ (PDB: 6KVP) centered on the allosteric ligand site with Vina through Monte Carlo algorithm [38]. The side chain of amino acids Met98, Val129, Ile197, Leu200, Val203, Asn208, Leu209, Val214, Met218, Ile228, Asn263, Val297, and Thr309 was set as flexible. Visualization was performed with PyMOL.

## 4. Conclusions

A collection of 15 new analogs of tripartite benzamide FtsZ inhibitors, varying in the nature of the central heterocyclic motif, was synthesized and evaluated against three *S. aureus* strains. It was found that the activities were correlated to the nature of the central heterocycle and also to the overall hydrophobicity of the compounds. Compound **II.c** with a central 1,2,4-oxadiazole ring, was found to be the most active in this study with MIC values of 0.5 µg/mL (for methicillin-sensitive and daptomycin-resistant *S. aureus*) and 1 µg/mL (for methicillin-resistant *S. aureus*). This compound is also deprived of any toxicity and induces an abnormal bacterial morphology due to a wrongly localized division septum.

## Figures and Tables

**Table 1:** Minimum inhibitory concentrations of series **I**, **II**, **III,** and **IV** molecules against *S. aureus* strains.

Structure	Compound	R	cLogP	MIC (µg/mL)		
				ATCC 29213 ^a^	SF8300 ^b^	ST20171643 ^c^
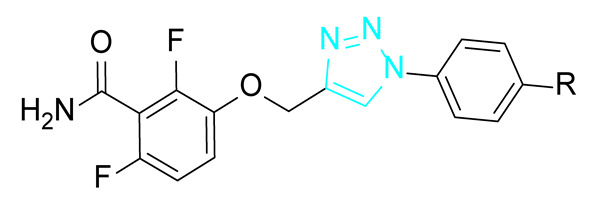	**I.a**	H	2.02	>256	>256	>256
	**I.b**	CF_3_	2.92	>256	>256	>256
	**I.c**	*t*-Bu	3.73	256	128	256
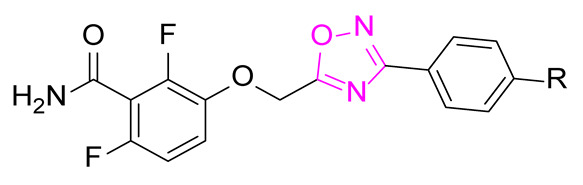	**II.a**	H	2.70	32	64	32
	**II.b**	CF_3_	3.60	16	32	8
	**II.c**	*t*-Bu	4.41	0.5	1	0.5
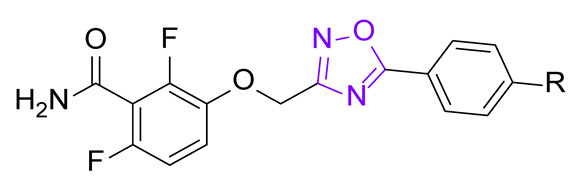	**III.a**	H	2.37	128	128	>256
	**III.b**	CF_3_	3.27	>256	>256	128
	**III.c**	*t*-Bu	4.08	8	8	8
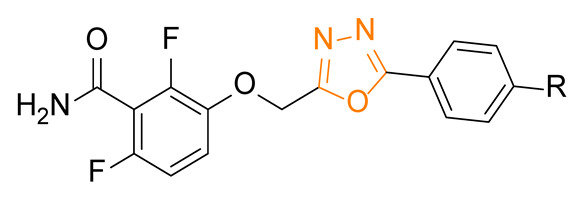	**IV.a**	H	2.01	>256	>256	>256
	**IV.b**	CF_3_	2.91	>256	>256	>256
	**IV.c**	*t*-Bu	3.72	256	256	256
	PC190723			0.5	0.5	0.5

^a^: Methicillin-sensitive *S. aureus*; ^b^: Methicillin-resistant *S. aureus*; ^c^: Daptomycin-resistant *S. aureus.*

**Table 2:** Minimum inhibitory concentrations of series **V** molecules against *S. aureus* strains.

Structure	Compound	R	cLogP	MIC (µg/mL)		
				ATCC 29213 ^a^	SF8300 ^b^	ST20171643 ^c^
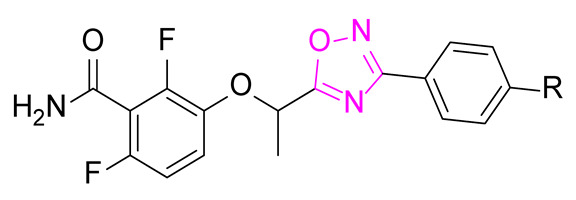	**V.a**	H	3.06	16	16	16
	**V.b**	CF_3_	3.96	1	1	1
	**V.c**	*t*-Bu	4.77	1	1	1
	PC190723			0.5	0.5	0.5

^a^: Methicillin-sensitive *S. aureus*; ^b^: Methicillin-resistant *S. aureus*; ^c^: Daptomycin-resistant *S. aureus.*

## Data Availability

Data can be found in the manuscript and in the Supplementary Materials.

## Supplementary Materials

The following supporting information can be downloaded at: https://www.mdpi.com/article/10.3390/molecules27196619/s1. Figure S1 (fluorescence microscope images with SF8300 bacteria), Figure S2 (docking studies of benzodioxane derivative), Figure S3 (docking studies of both enantiomers of compounds **V.b** and **V.c**) and copies of 1H and 13C NMR spectra of final products are available in supplementary information.
