# Supplementary material for: Structural Variations in the Central Heterocyclic Scaffold of Tripartite 2,6-Difluorobenzamides: Influence on Their Antibacterial Activity against MDR Staphylococcus aureus
Source: Molecules. 2022 Oct 5;27(19):6619. doi: 10.3390/molecules27196619 (PMC9573484; doi:10.3390/molecules27196619)
Supplement: Supplementary file 1 [file molecules-27-06619-s001.zip › molecules-1945323-supplementary.pdf]

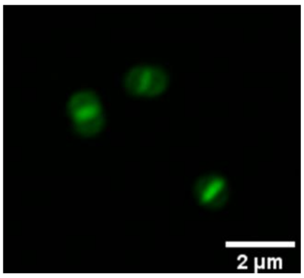

Negative control

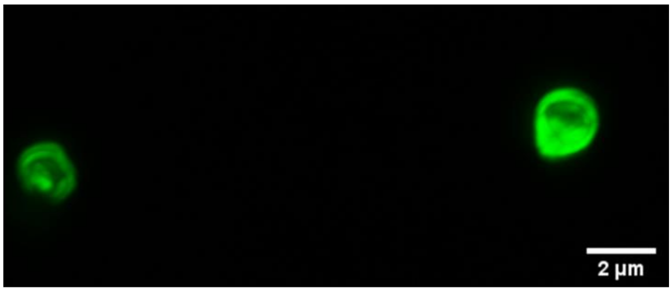

PC190723 (2 µg/mL)

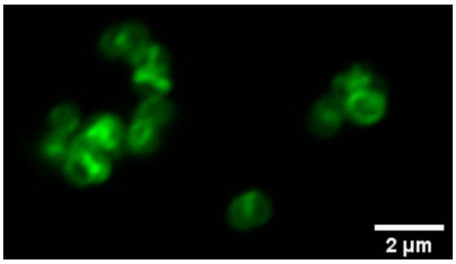

II.c (1 µg/mL)

Figure S1. Fluorescence microscope images of SF8300 bacteria stained with vancomycin-bodipy conjugate in presence or absence of antibacterial agent.

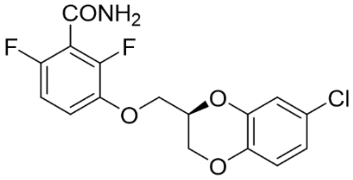

Benzodioxane 1

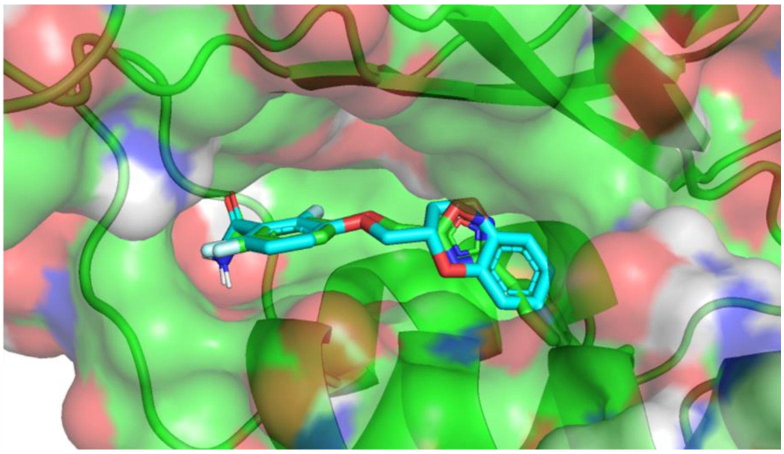

Figure S2: Superimposition of docking results of compound **II.c** (green) and **benzodioxane 1** (cyan) within the allosteric binding site of FtsZ.

Compound	**V.b**	
Enantiomer	(R)	(S)
Structure	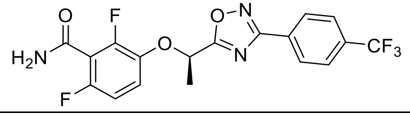	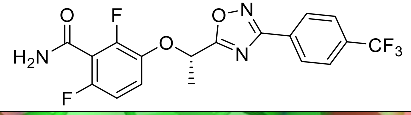
Docking and superimposition with co-crystallized inhibitor	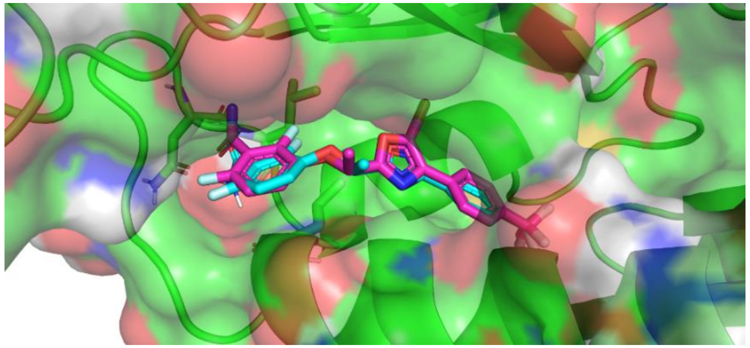	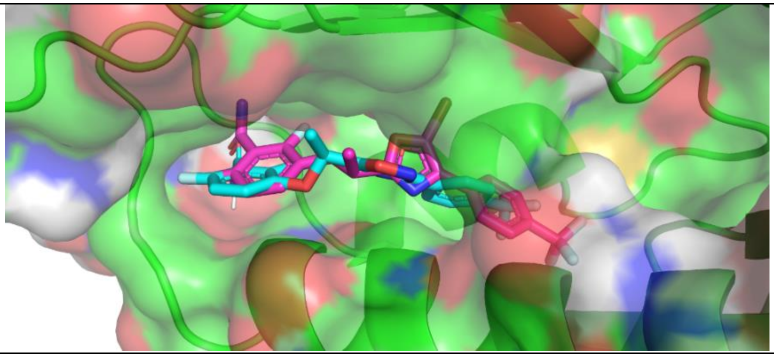

Compound	**V.c**	
Enantiomer	(R)	(S)
Structure	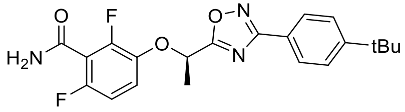	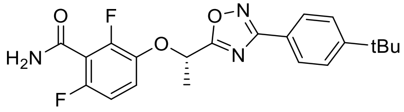
Docking and superimposition with co-crystallized inhibitor	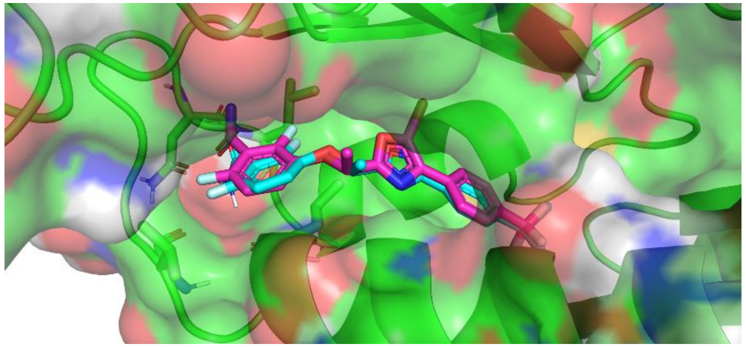	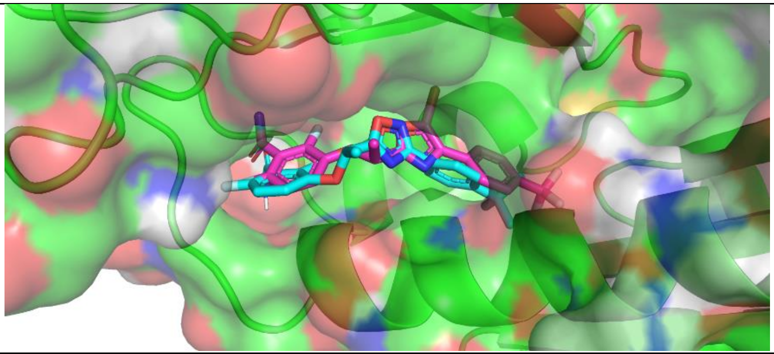

Figure S3: Docking of both enantiomers of compound **V.b** and **V.c** within the allosteric binding site of FtsZ (PDB code: 6KVP) [6] . Docking results (cyan) are superimposed with the co-crystallized oxazole inhibitor (magenta).

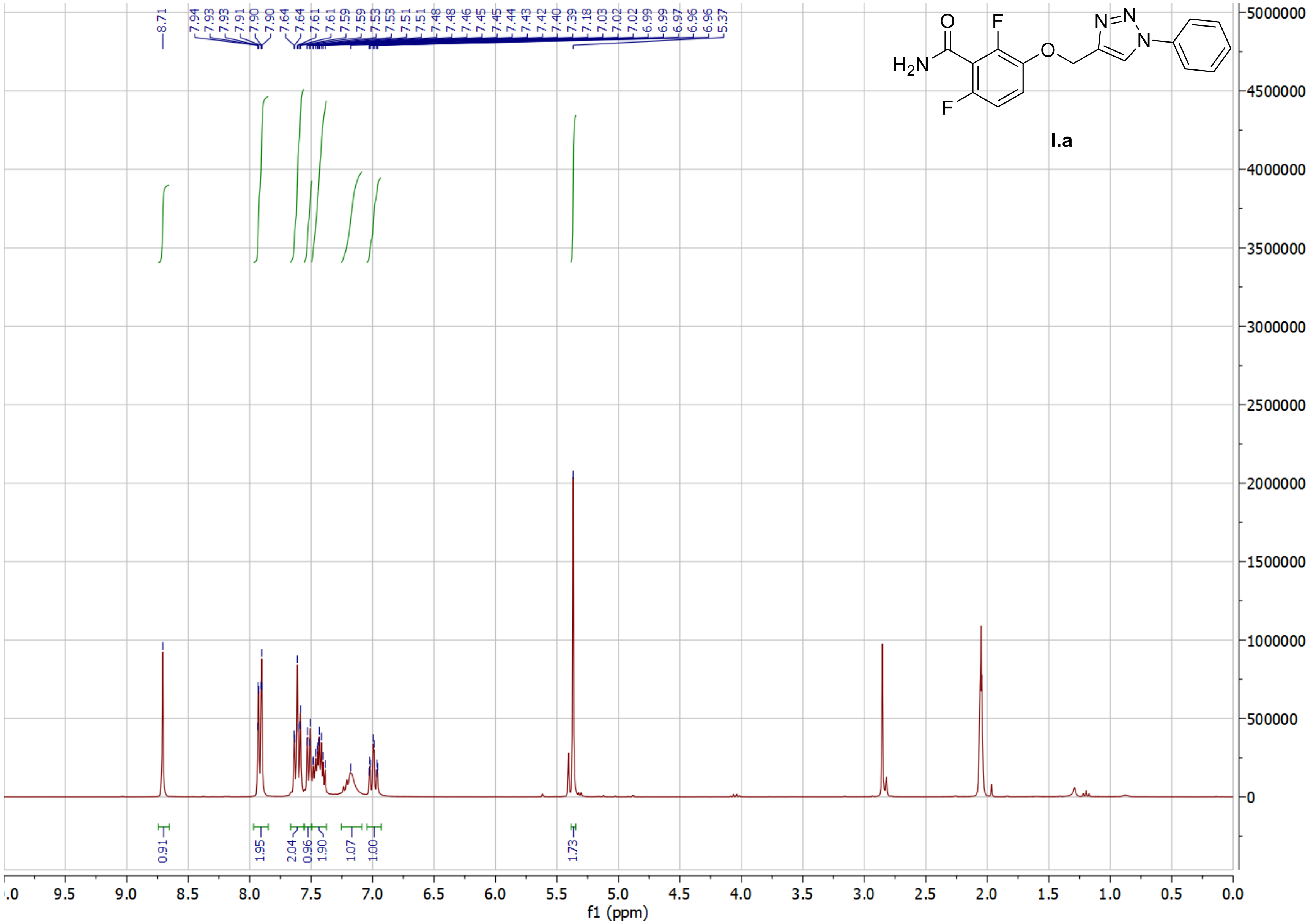
8.71
7.94
7.93
7.93
7.91
7.90
7.90
7.64
7.64
7.61
7.61
7.59
7.59
7.53
7.53
7.51
7.51
7.48
7.48
7.46
7.45
7.45
7.44
7.43
7.42
7.40
7.39
7.18
7.03
7.02
7.02
6.99
6.97
6.96
6.96
5.37
O
F
N=N
H2N
O
N
F
I.a
0.91
1.95
2.04
0.96
1.90
1.07
1.00
1.73
5000000
4500000
4000000
3500000
3000000
2500000
2000000
1500000
1000000
500000
0
9.5
9.0
8.5
8.0
7.5
7.0
6.5
6.0
5.5
5.0
4.5
4.0
3.5
3.0
2.5
2.0
1.5
1.0
0.5
0.0
f1 (ppm)

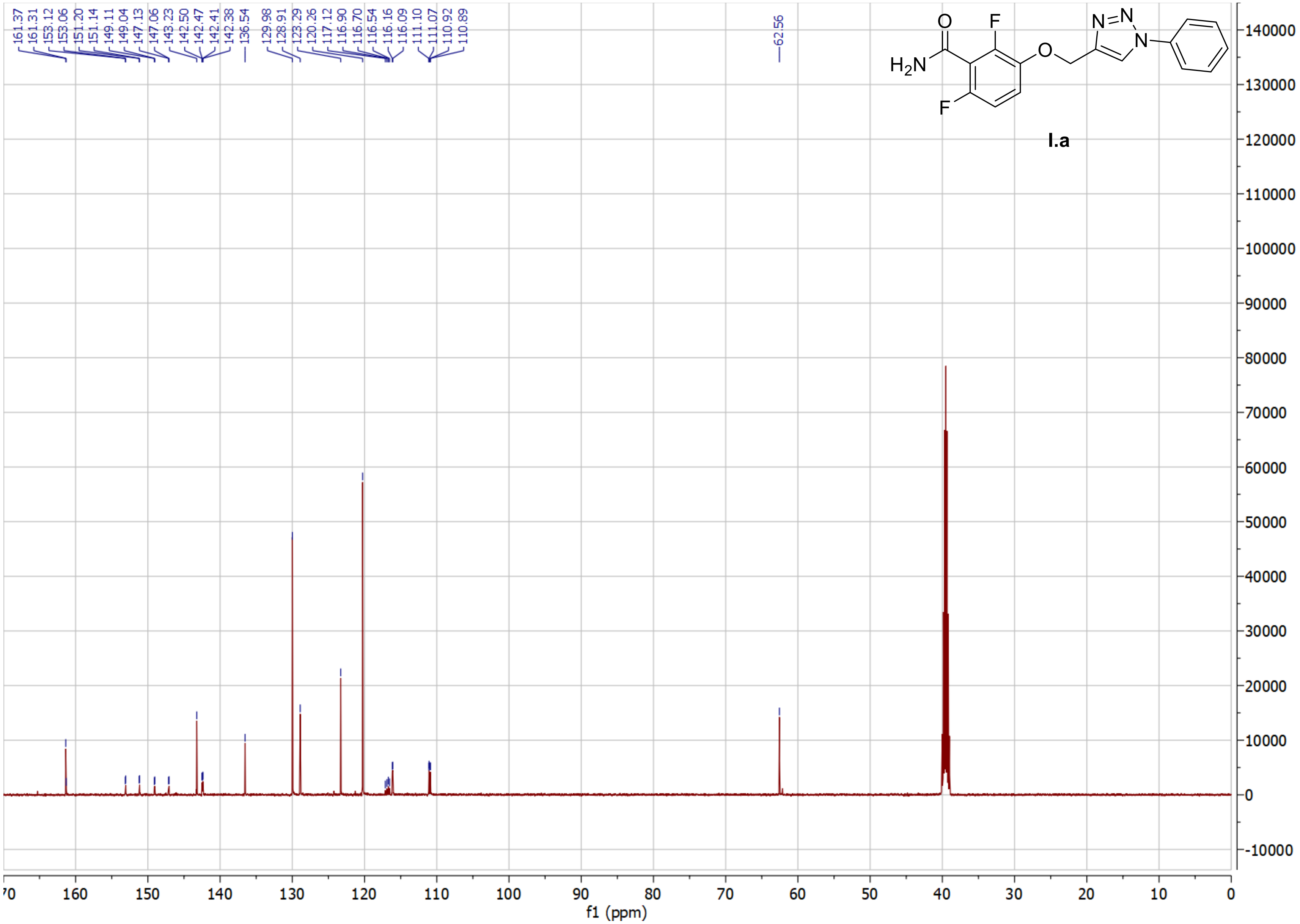

161.37
161.31
153.12
153.06
151.20
151.14
149.11
149.04
147.13
147.06
143.23
142.50
142.47
142.41
142.38
136.54
129.98
128.91
123.29
120.26
117.12
116.90
116.70
116.54
116.16
116.09
111.10
111.07
110.92
110.89
62.56
I.a
f1 (ppm)

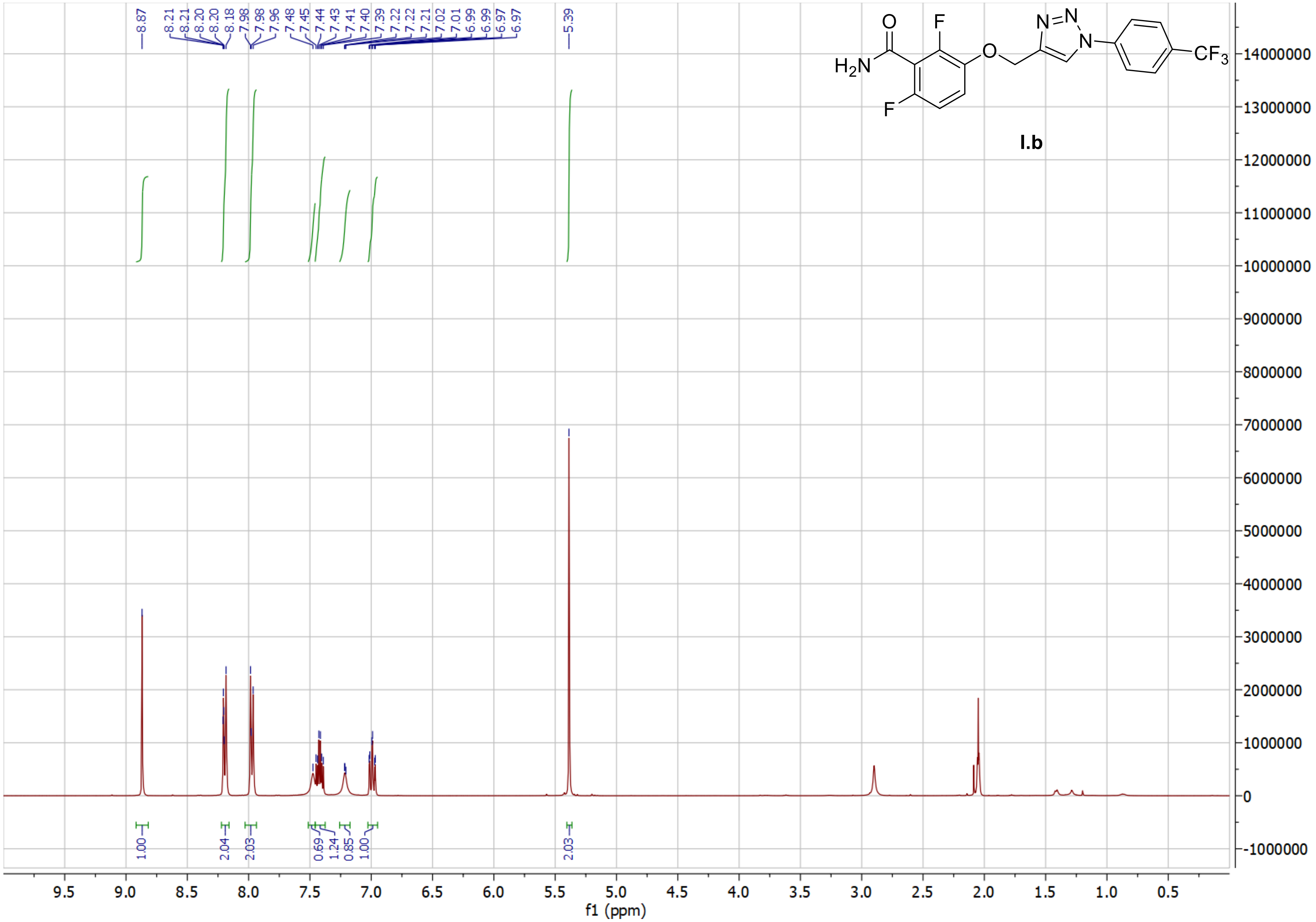
8.87
8.21
8.21
8.20
8.20
8.18
7.98
7.98
7.96
7.48
7.45
7.44
7.43
7.41
7.40
7.39
7.22
7.22
7.21
7.02
7.01
6.99
6.99
6.97
6.97
5.39
I.b
1.00
2.04
2.03
0.69
1.24
0.85
1.00
2.03
f1 (ppm)

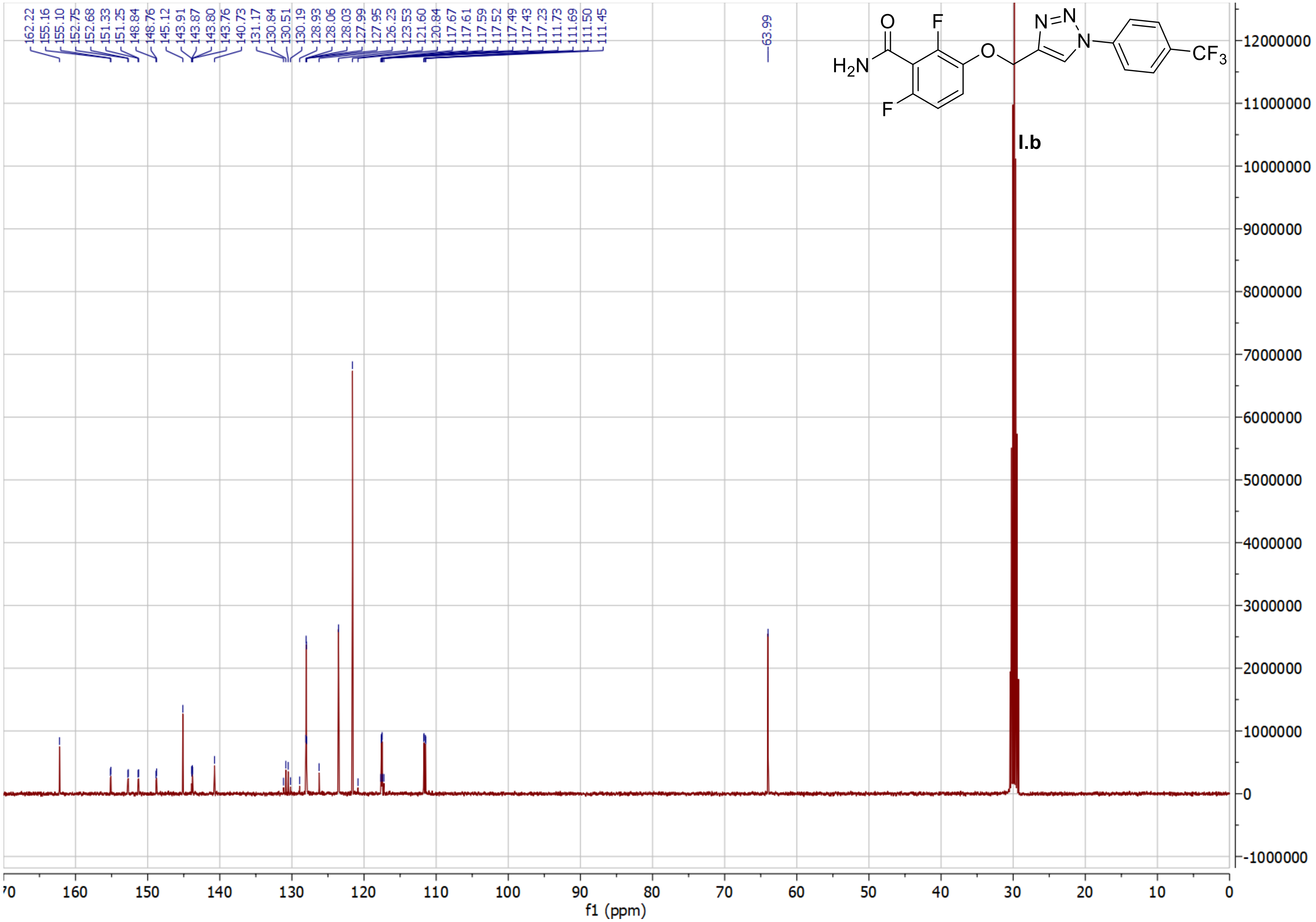
162.22
155.16
155.10
152.75
152.68
151.33
151.25
148.84
148.76
145.12
143.91
143.87
143.80
143.76
140.73
131.17
130.84
130.51
130.19
128.93
128.06
128.03
127.99
127.95
126.23
123.53
121.60
120.84
117.67
117.61
117.59
117.52
117.49
117.43
117.23
111.73
111.69
111.50
111.45
63.99
I.b
f1 (ppm)

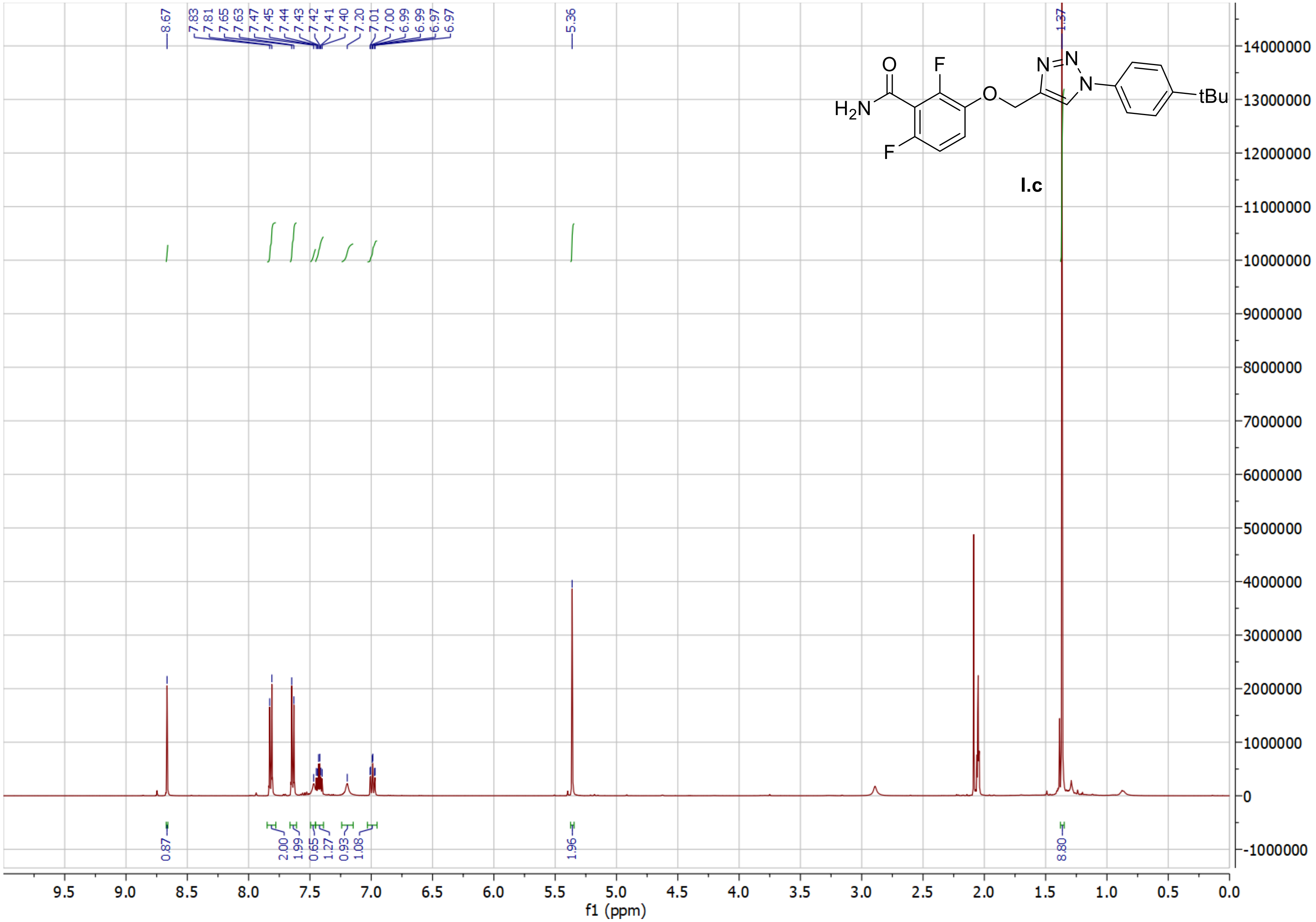

8.67
7.83
7.81
7.65
7.63
7.47
7.45
7.44
7.43
7.42
7.41
7.40
7.20
7.01
7.00
6.99
6.99
6.97
6.97
5.36
1.37
O
F
N
N
N
O
H2N
tBu
F
I.c
0.87
2.00
1.99
0.65
1.27
0.93
1.08
1.96
8.80
14000000
13000000
12000000
11000000
10000000
9000000
8000000
7000000
6000000
5000000
4000000
3000000
2000000
1000000
0
-1000000
9.5
9.0
8.5
8.0
7.5
7.0
6.5
6.0
5.5
5.0
4.5
4.0
3.5
3.0
2.5
2.0
1.5
1.0
0.5
0.0
f1 (ppm)

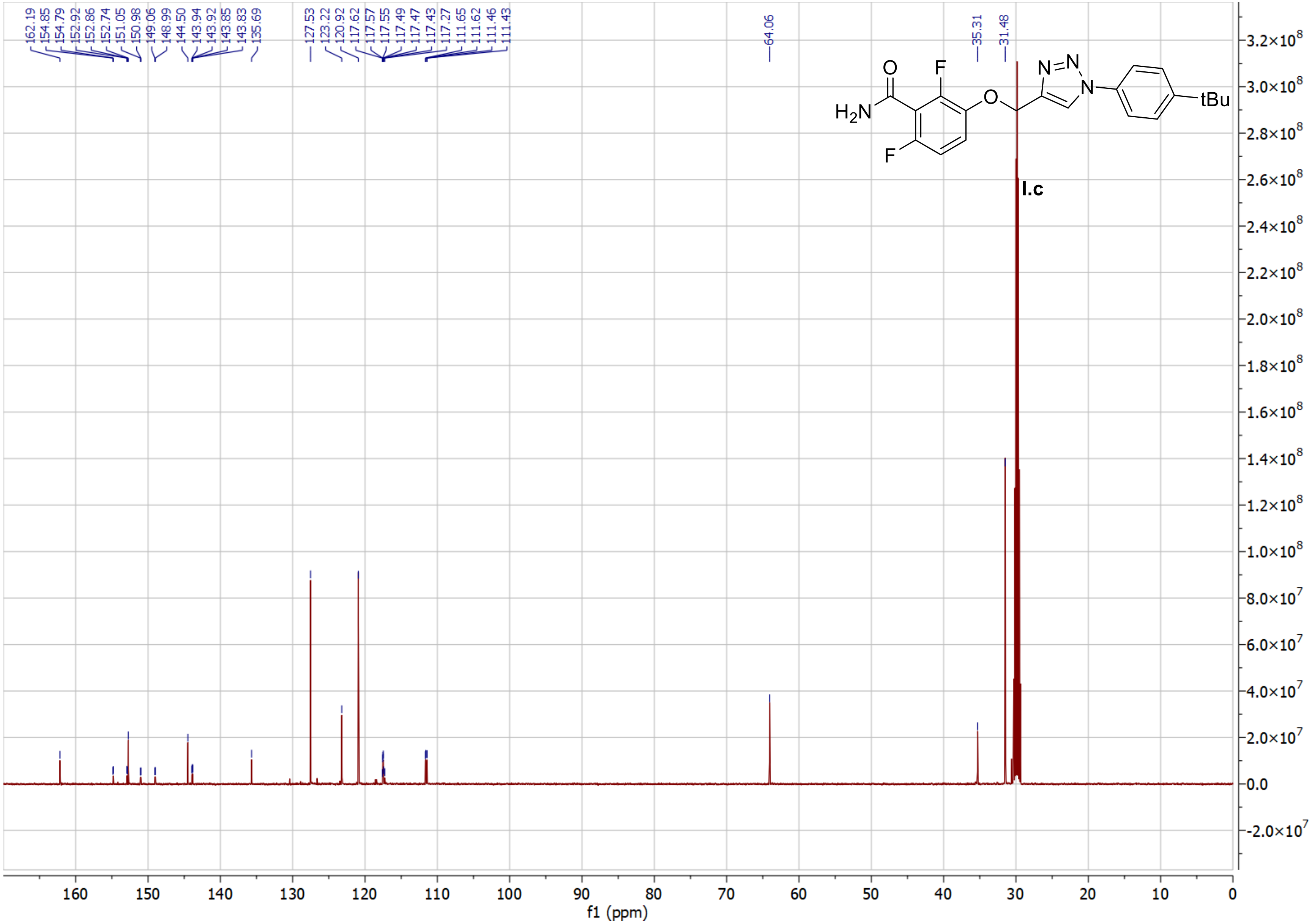

162.19
154.85
154.79
152.92
152.86
152.74
151.05
150.98
149.06
148.99
144.50
143.94
143.92
143.85
143.83
135.69
127.53
123.22
120.92
117.62
117.57
117.55
117.49
117.47
117.43
117.27
111.65
111.62
111.46
111.43
64.06
35.31
31.48
I.c
tBu
f1 (ppm)

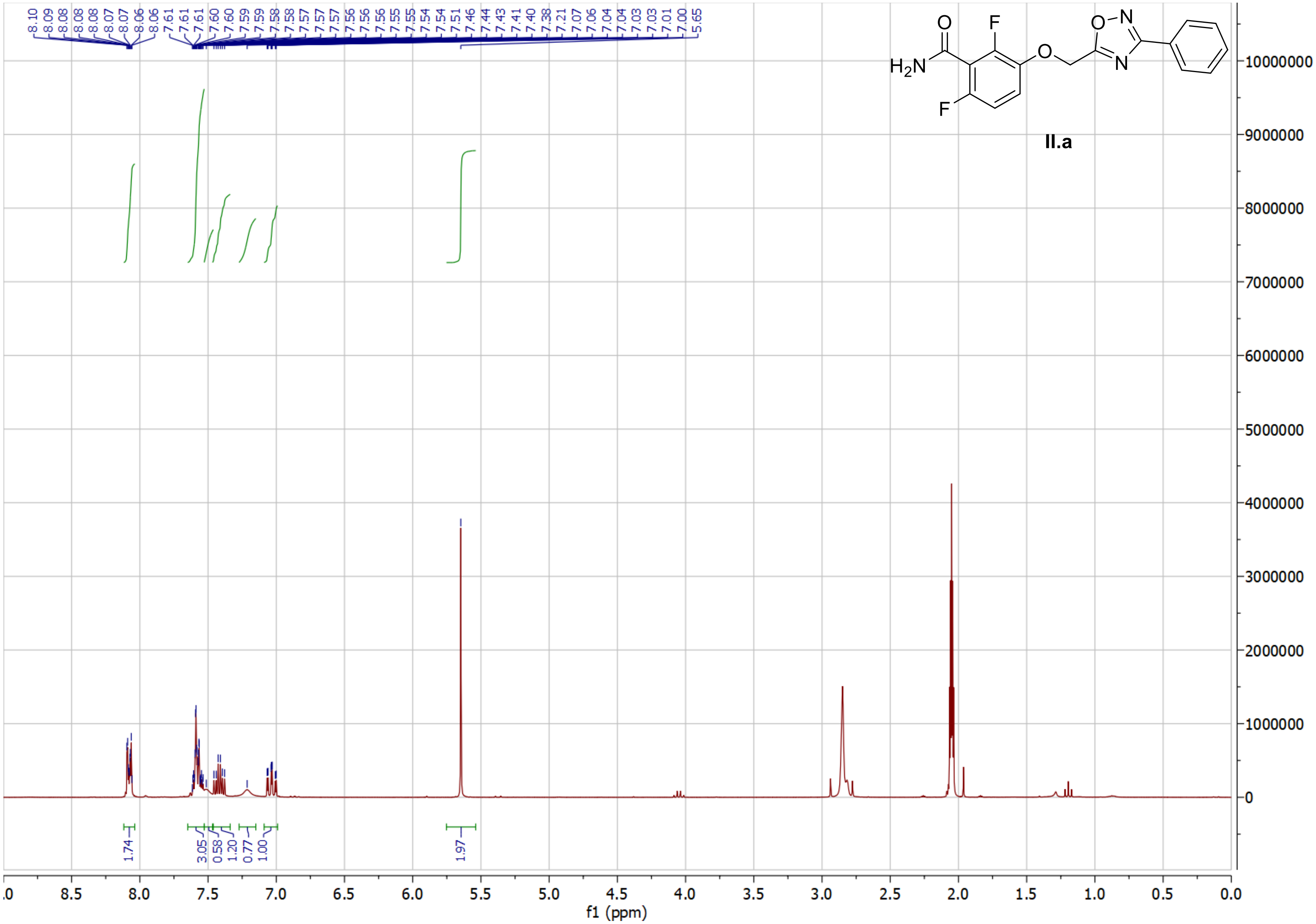

8.10 8.09 8.08 8.08 8.07 8.06 8.06 7.61 7.61 7.61 7.60 7.60 7.59 7.59 7.58 7.58 7.57 7.57 7.57 7.56 7.56 7.56 7.55 7.55 7.54 7.54 7.51 7.46 7.44 7.43 7.41 7.40 7.38 7.21 7.07 7.06 7.04 7.04 7.03 7.03 7.01 7.00 5.65
O
F
O
O–N
N
H2N
F
II.a
1.74
3.05
0.58
1.20
0.77
1.00
1.97
f1 (ppm)

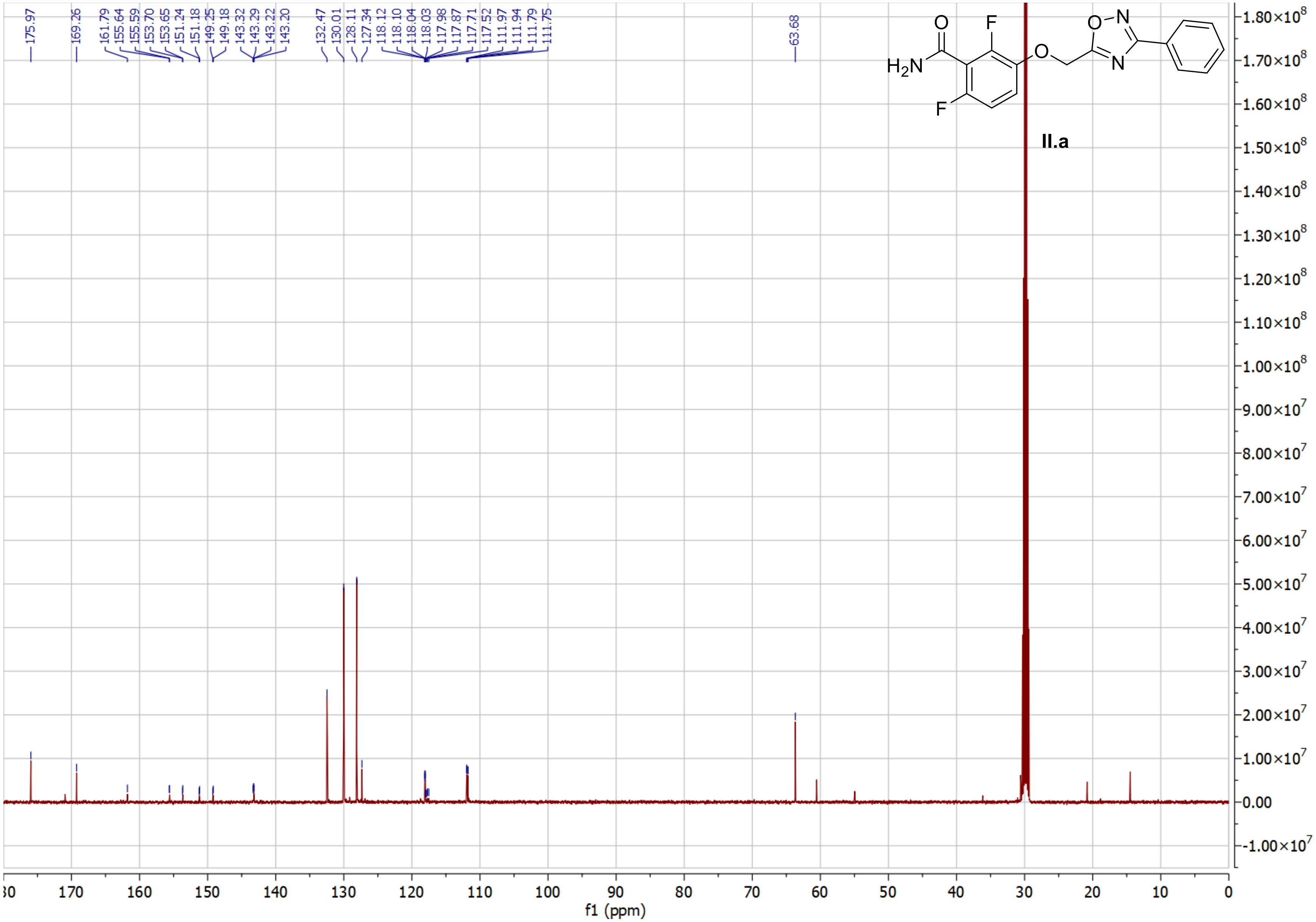
175.97
169.26
161.79
155.64
155.59
153.70
153.65
151.24
151.18
149.25
149.18
143.32
143.29
143.22
143.20
132.47
130.01
128.11
127.34
118.12
118.10
118.04
118.03
117.98
117.87
117.71
117.52
111.97
111.94
111.79
111.75
63.68
II.a
f1 (ppm)

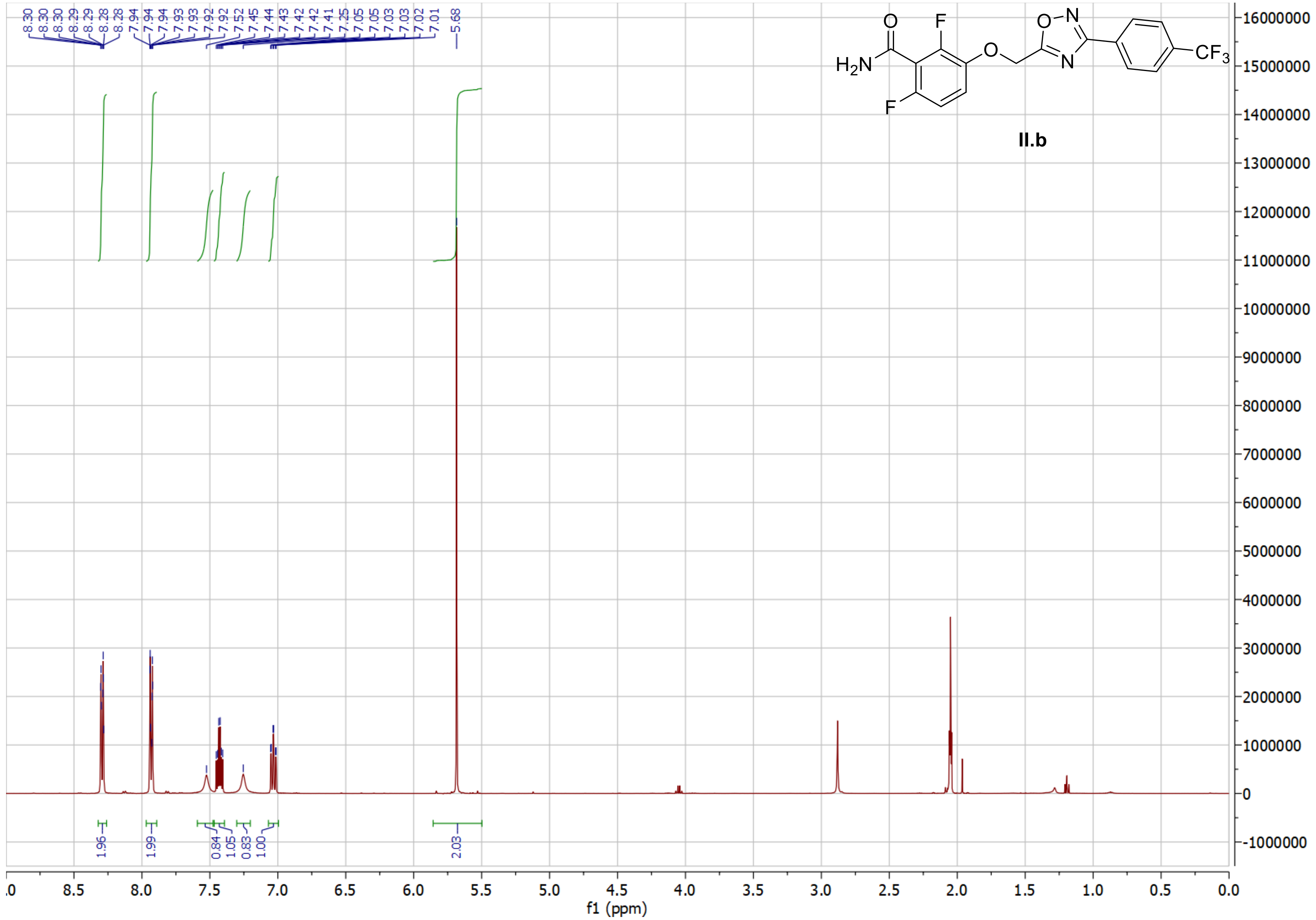

8.30
8.30
8.30
8.29
8.29
8.28
8.28
7.94
7.94
7.94
7.93
7.93
7.92
7.92
7.52
7.45
7.44
7.43
7.42
7.42
7.41
7.25
7.05
7.05
7.03
7.03
7.02
7.01
5.68
1.96
1.99
0.84
1.05
0.83
1.00
2.03
f1 (ppm)
II.b

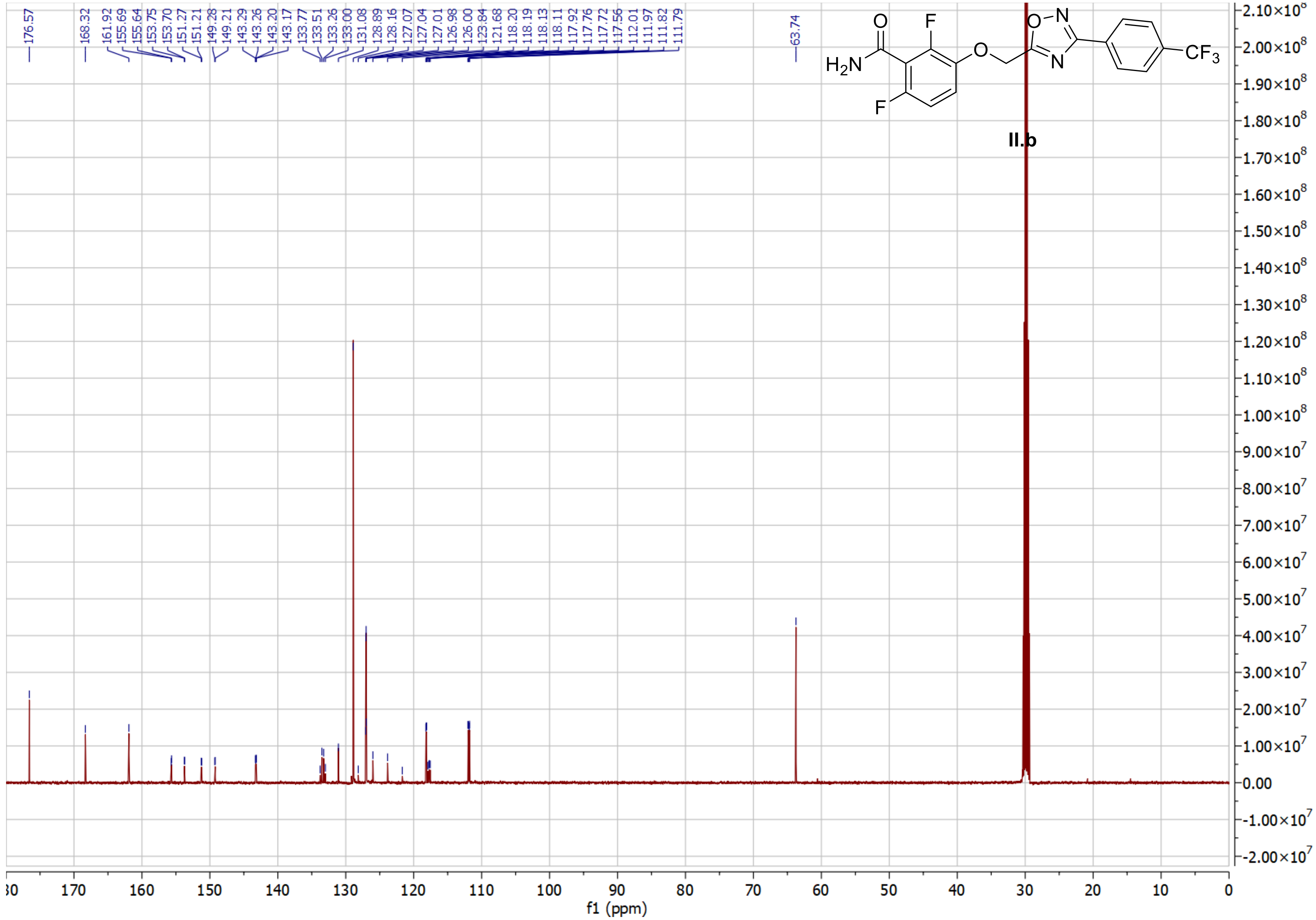
176.57
168.32
161.92
155.69
155.64
153.75
153.70
151.27
151.21
149.28
149.21
143.29
143.26
143.20
143.17
133.77
133.51
133.26
133.00
131.08
128.89
128.16
127.07
127.04
127.01
126.98
126.00
123.84
121.68
118.20
118.19
118.13
118.11
117.92
117.76
117.72
117.56
112.01
111.97
111.82
111.79
63.74
II.b
f1 (ppm)

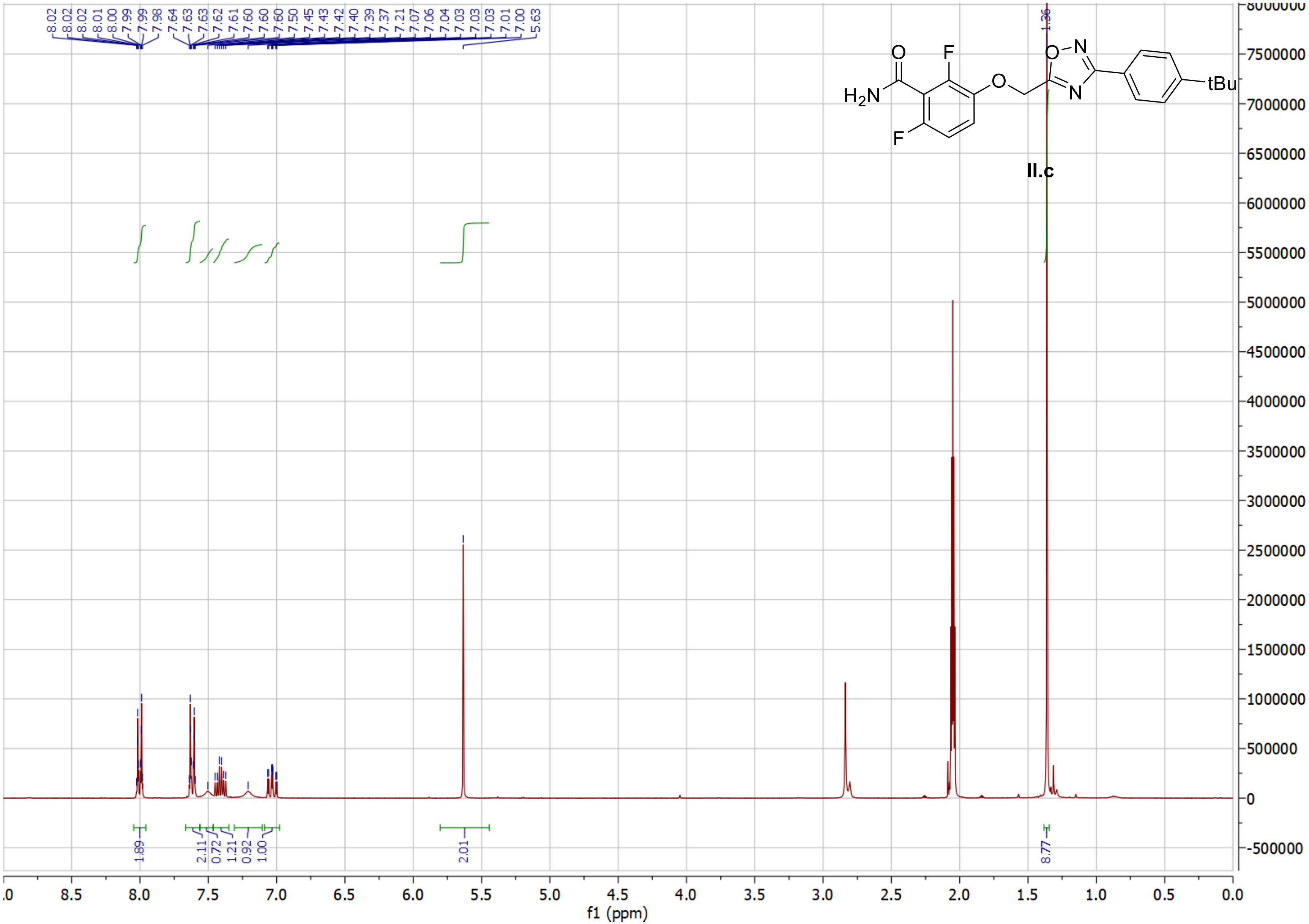

8.02 8.02 8.02 8.01 8.00 7.99 7.99 7.98 7.64 7.63 7.63 7.62 7.61 7.60 7.60 7.60 7.50 7.45 7.43 7.42 7.40 7.39 7.37 7.21 7.07 7.06 7.04 7.03 7.03 7.03 7.01 7.00 5.63
1.36
O
F
H2N
O
O-N
N
tBu
F
II.c
1.89
2.11
0.72
1.21
0.92
1.00
2.01
8.77
f1 (ppm)

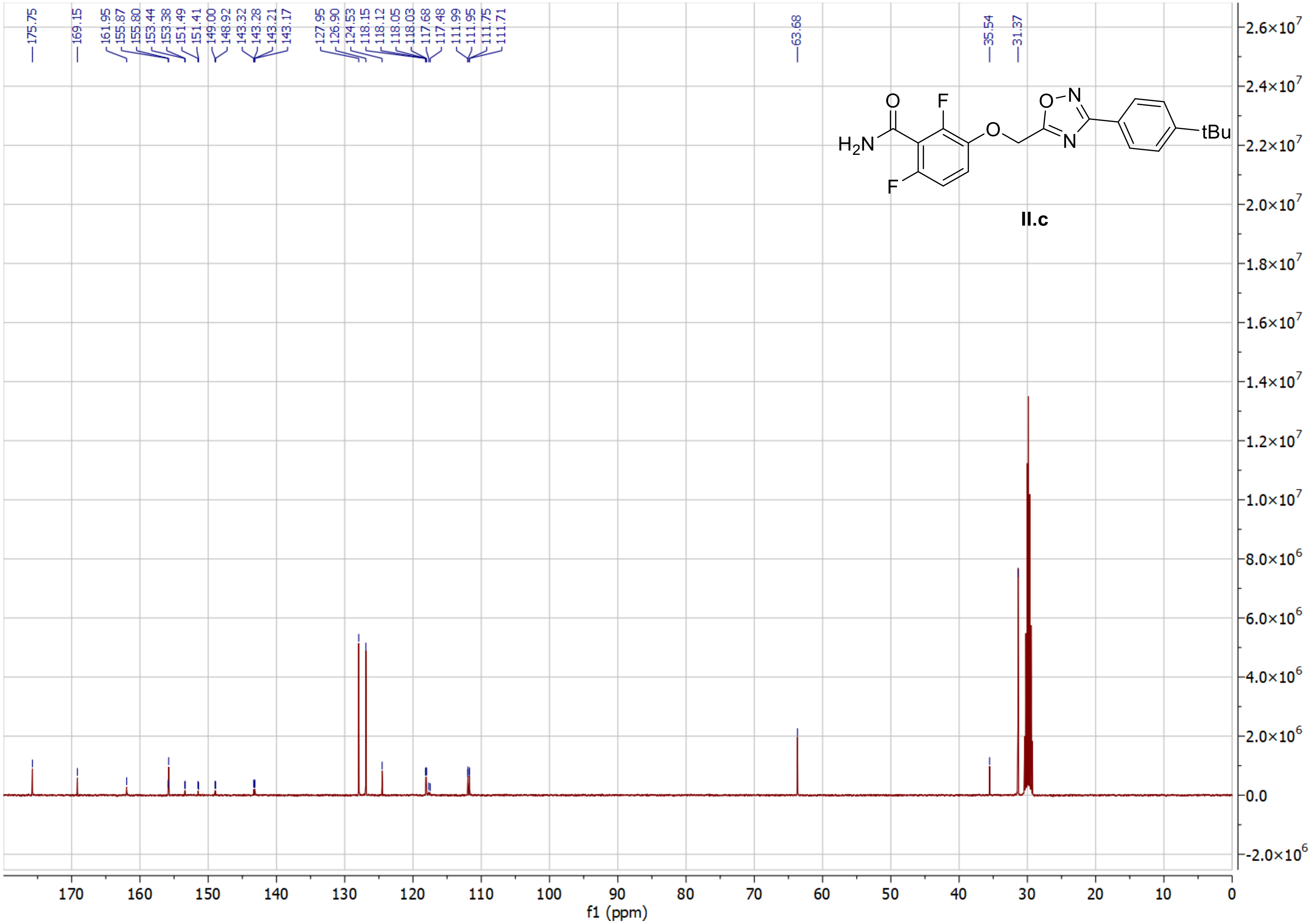
175.75
169.15
161.95
155.87
155.80
153.44
153.38
151.49
151.41
149.00
148.92
143.32
143.28
143.21
143.17
127.95
126.90
124.53
118.15
118.12
118.05
118.03
117.68
117.48
111.99
111.95
111.75
111.71
63.68
35.54
31.37
H2N
O
F
F
O
O
N
N
tBu
II.c
f1 (ppm)

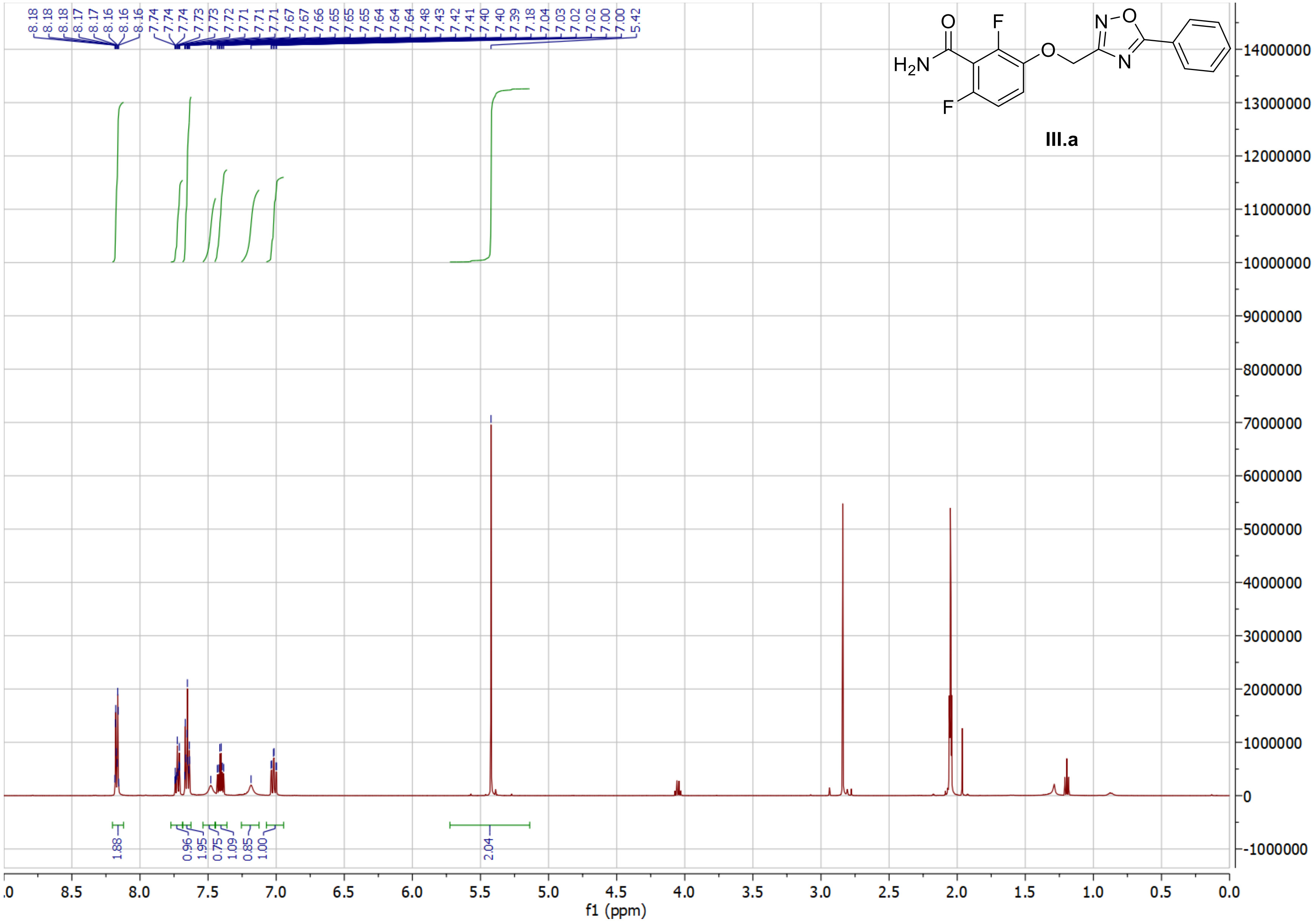
8.18 8.18 8.18 8.17 8.17 8.16 8.16 8.16 7.74 7.74 7.74 7.73 7.73 7.72 7.71 7.71 7.71 7.67 7.67 7.66 7.65 7.65 7.65 7.64 7.64 7.64 7.48 7.43 7.42 7.41 7.40 7.40 7.39 7.18 7.04 7.03 7.02 7.02 7.00 7.00 5.42
III.a
1.88
0.96
1.95
0.75
1.09
0.85
1.00
2.04
f1 (ppm)

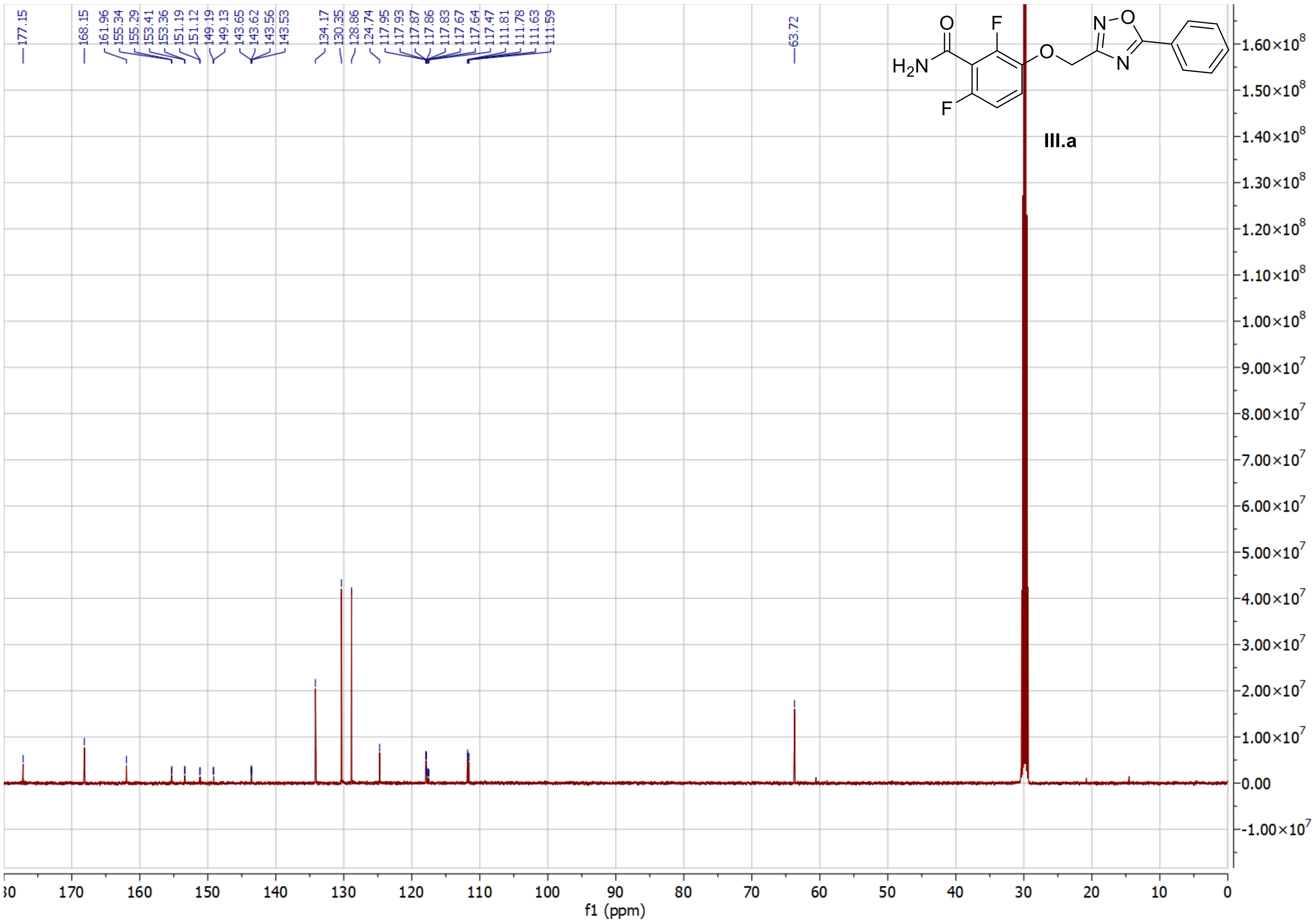

177.15
168.15
161.96
155.34
155.29
153.41
153.36
151.19
151.12
149.19
149.13
143.65
143.62
143.56
143.53
134.17
130.35
128.86
124.74
117.95
117.93
117.87
117.86
117.83
117.67
117.64
117.47
111.81
111.78
111.63
111.59
63.72
O
F
H2N
F
O
N
O
N
III.a
1.60×10^8
1.50×10^8
1.40×10^8
1.30×10^8
1.20×10^8
1.10×10^8
1.00×10^8
9.00×10^7
8.00×10^7
7.00×10^7
6.00×10^7
5.00×10^7
4.00×10^7
3.00×10^7
2.00×10^7
1.00×10^7
0.00
-1.00×10^7
170 160 150 140 130 120 110 100 90 80 70 60 50 40 30 20 10 0
f1 (ppm)

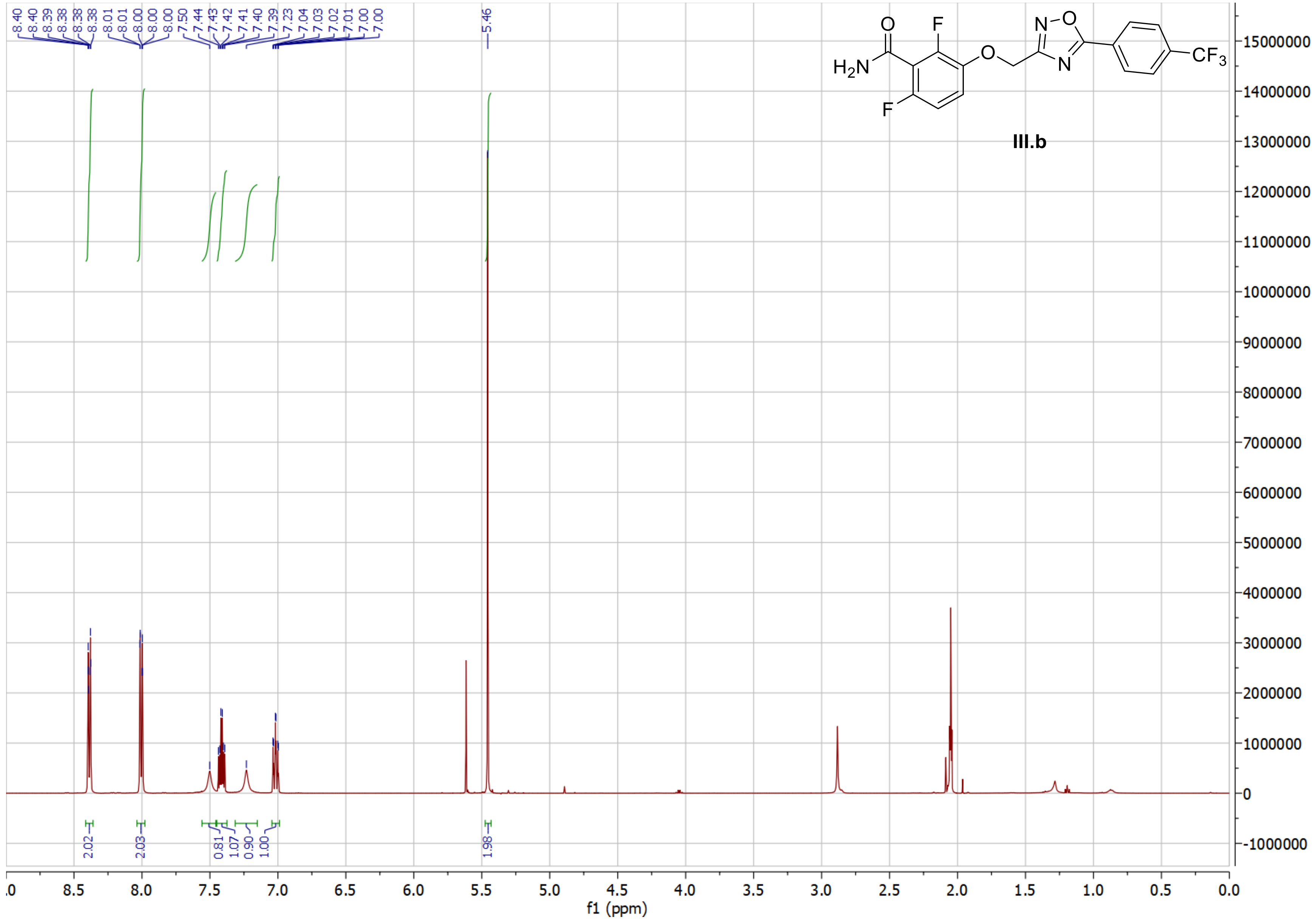
8.40
8.40
8.39
8.38
8.38
8.38
8.01
8.01
8.00
8.00
7.50
7.44
7.43
7.42
7.41
7.40
7.39
7.23
7.04
7.03
7.02
7.01
7.00
7.00
5.46
2.02
2.03
0.81
1.07
0.90
1.00
1.98
f1 (ppm)
III.b

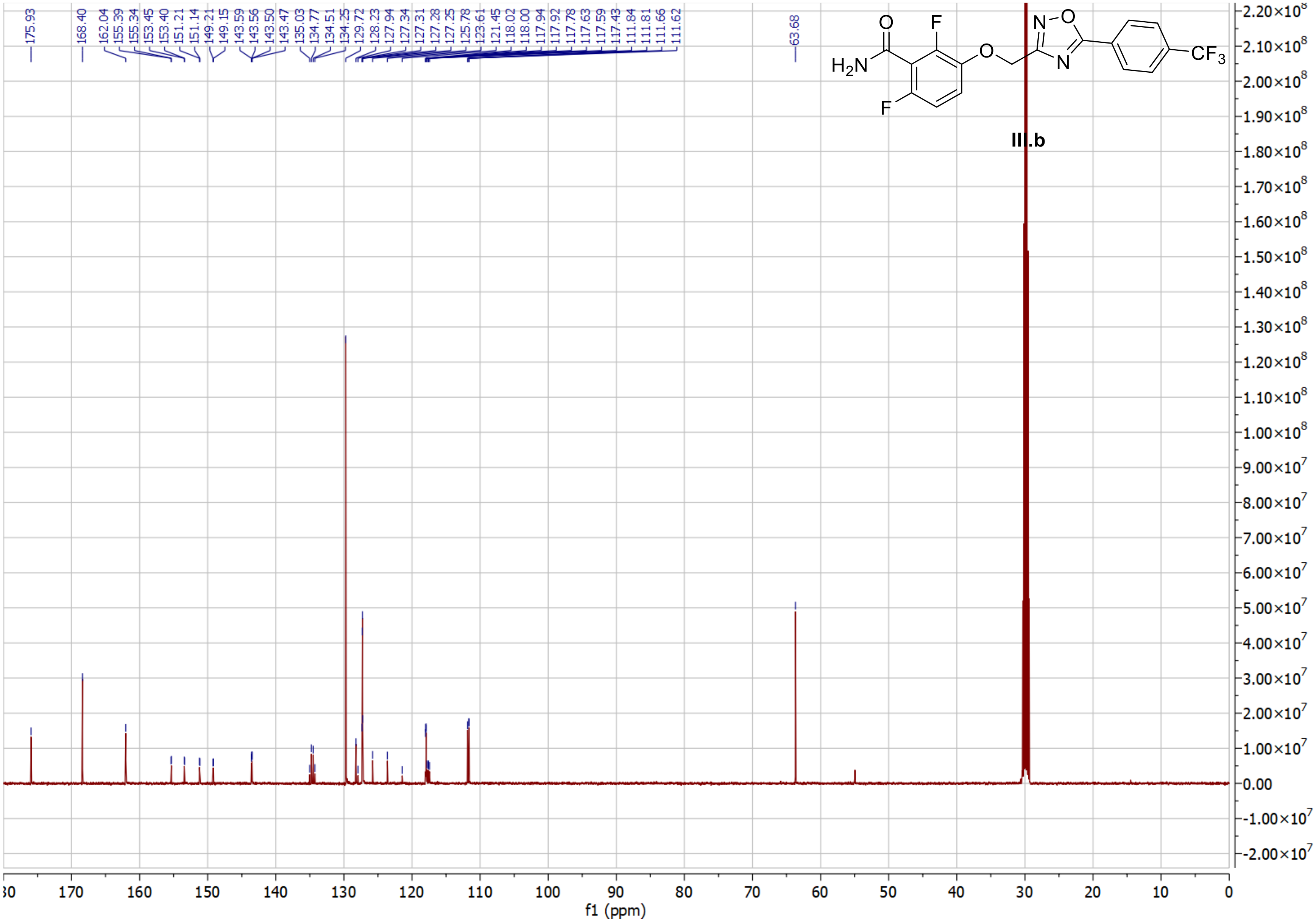
175.93
168.40
162.04
155.39
155.34
153.45
153.40
151.21
151.14
149.21
149.15
143.59
143.56
143.50
143.47
135.03
134.77
134.51
134.25
129.72
128.23
127.94
127.34
127.31
127.28
127.25
125.78
123.61
121.45
118.02
118.00
117.94
117.92
117.78
117.63
117.59
117.43
111.84
111.81
111.66
111.62
63.68
III.b
f1 (ppm)

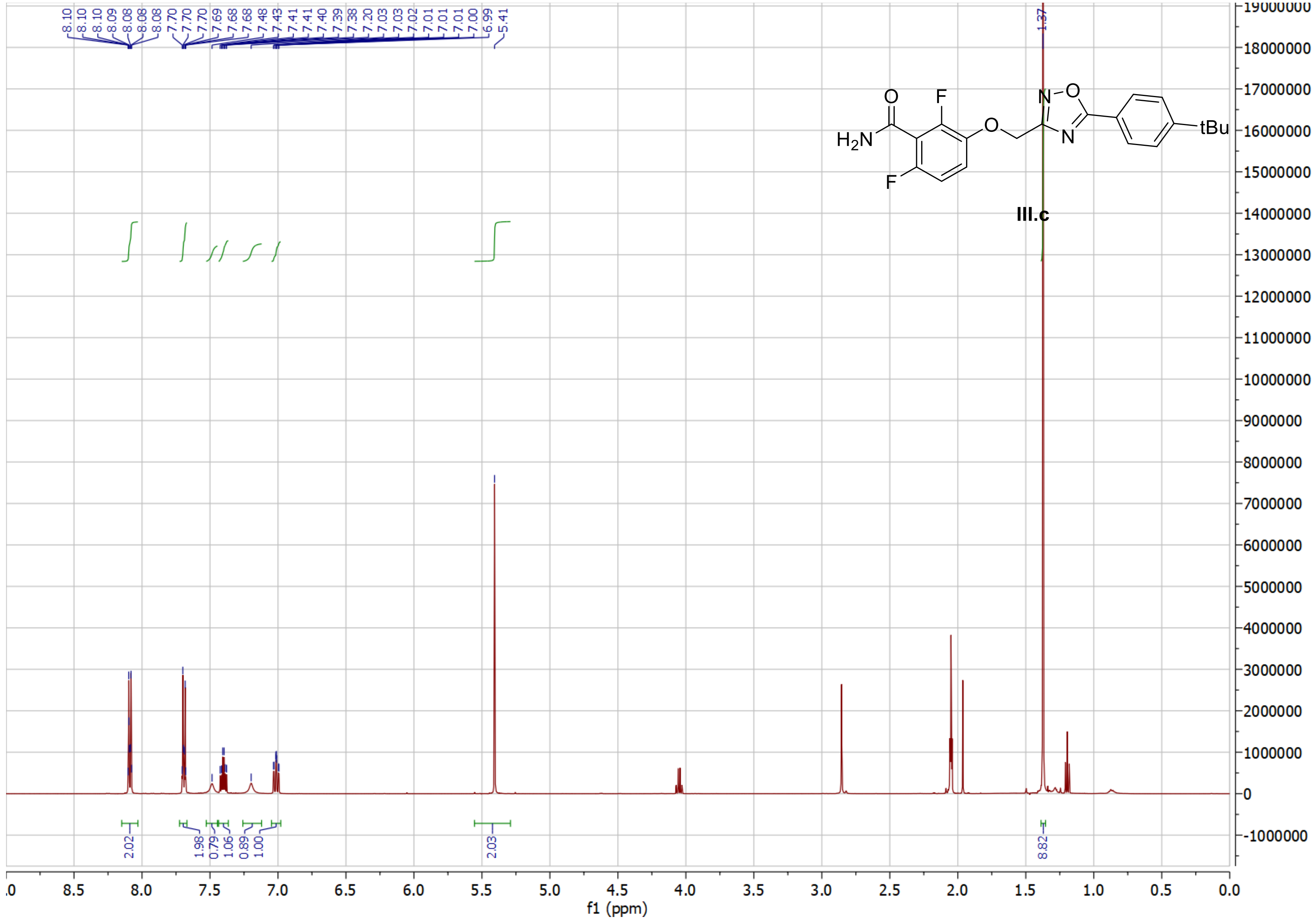
8.10
8.10
8.10
8.09
8.08
8.08
8.08
7.70
7.70
7.70
7.69
7.68
7.68
7.48
7.43
7.41
7.41
7.40
7.39
7.38
7.20
7.03
7.03
7.02
7.01
7.01
7.01
7.00
6.99
5.41
1.37
2.02
1.98
0.79
1.06
0.89
1.00
2.03
8.82
H2N
O
F
F
O
N
O
N
tBu
III.c
f1 (ppm)

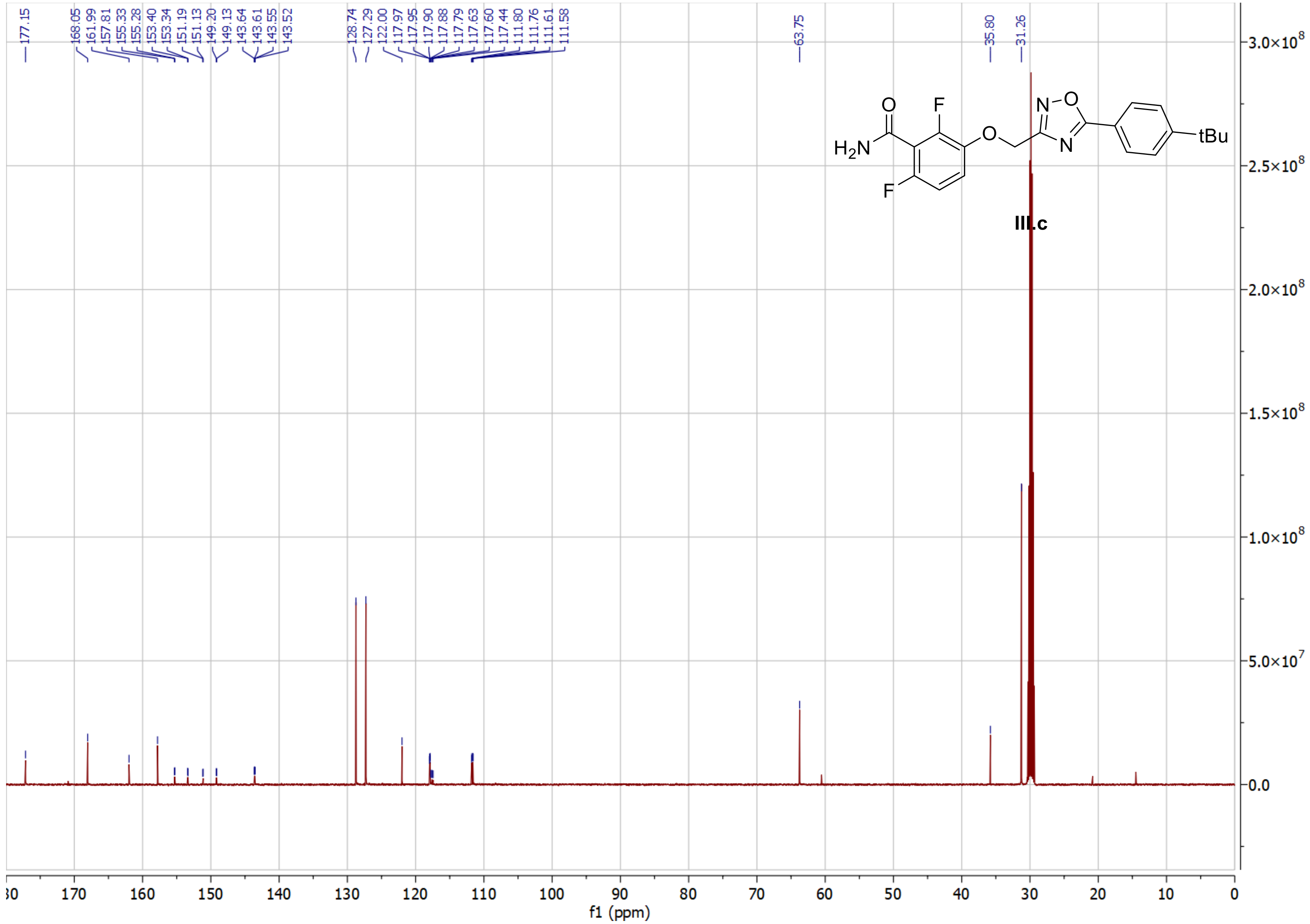
177.15
168.05
161.99
157.81
155.33
155.28
153.40
153.34
151.19
151.13
149.20
149.13
143.64
143.61
143.55
143.52
128.74
127.29
122.00
117.97
117.95
117.90
117.88
117.79
117.63
117.60
117.44
111.80
111.76
111.61
111.58
63.75
35.80
31.26
III.c
tBu
f1 (ppm)

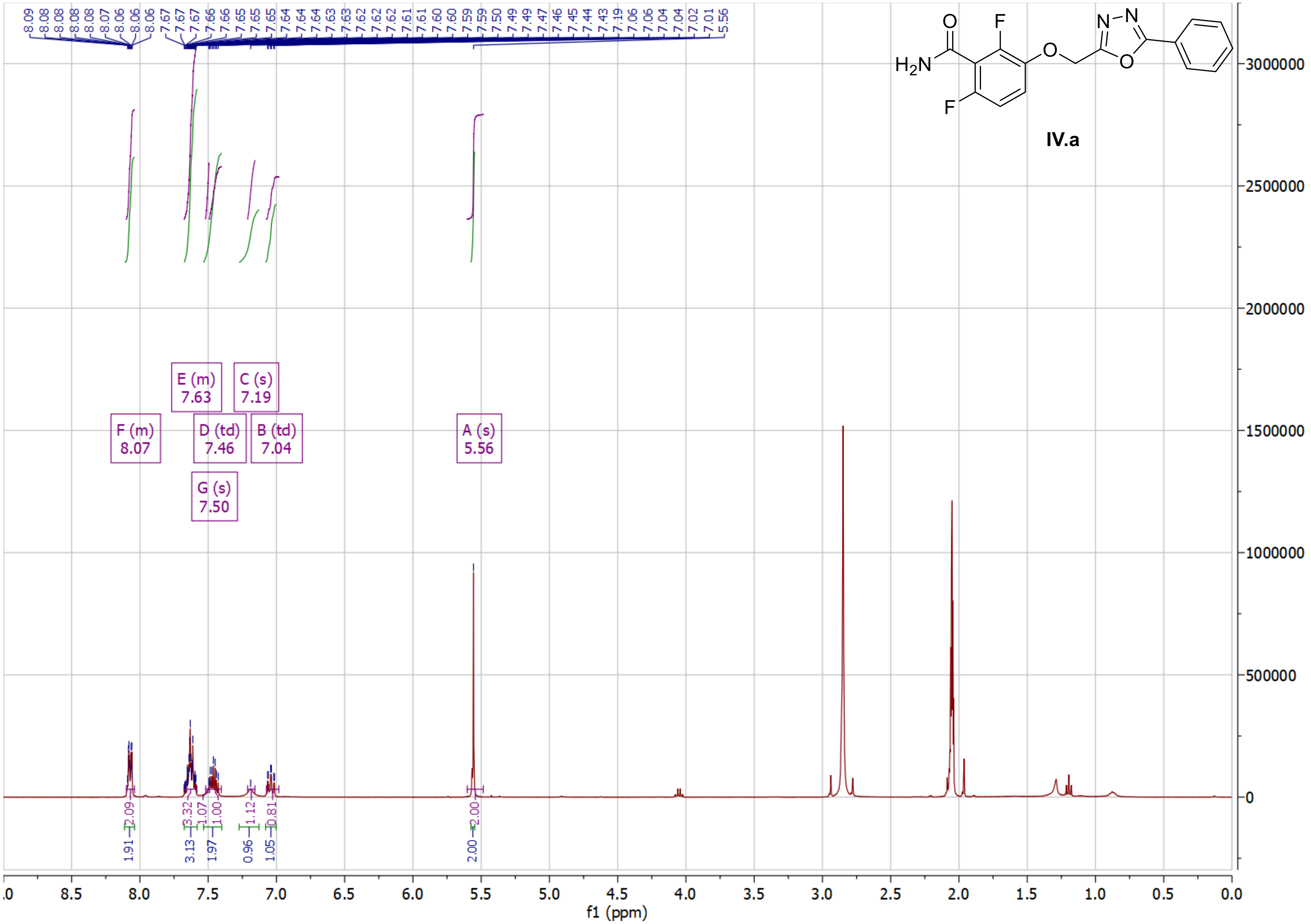

8.09 8.08 8.08 8.08 8.08 8.07 8.06 8.06 8.06 7.67 7.67 7.67 7.66 7.66 7.65 7.65 7.65 7.64 7.64 7.64 7.63 7.63 7.62 7.62 7.62 7.61 7.61 7.60 7.60 7.59 7.59 7.50 7.49 7.49 7.47 7.46 7.45 7.44 7.43 7.19 7.06 7.06 7.04 7.04 7.02 7.01 5.56
F (m) 8.07
E (m) 7.63
G (s) 7.50
D (td) 7.46
C (s) 7.19
B (td) 7.04
A (s) 5.56
2.09 3.32 1.07 1.00 1.12 0.81 2.00
1.91 3.13 1.97 0.96 1.05 2.00
f1 (ppm)
IV.a

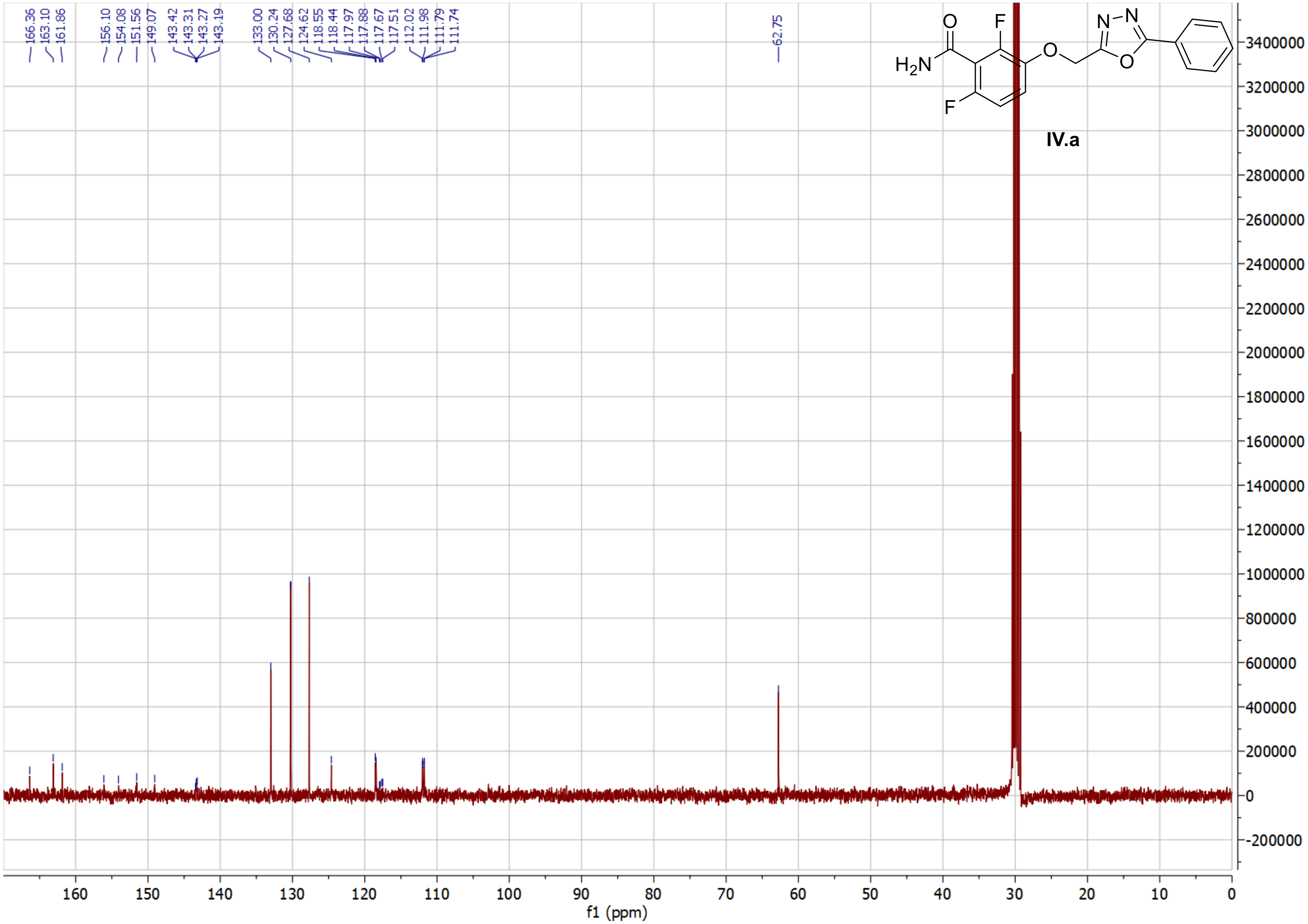
166.36
163.10
161.86
156.10
154.08
151.56
149.07
143.42
143.31
143.27
143.19
133.00
130.24
127.68
124.62
118.55
118.44
117.97
117.88
117.67
117.51
112.02
111.98
111.79
111.74
62.75
IV.a
f1 (ppm)

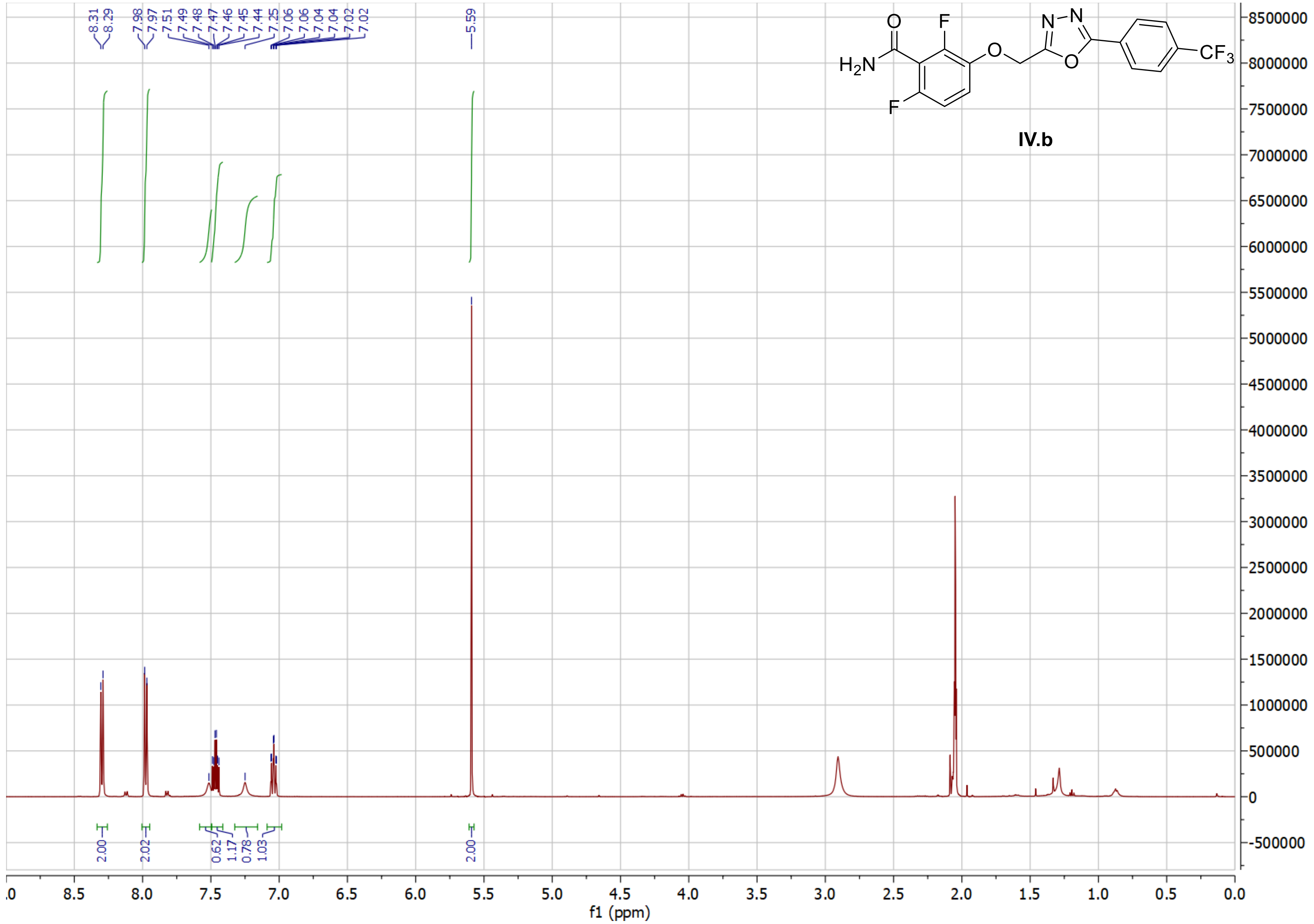

8.31
8.29
7.98
7.97
7.51
7.49
7.48
7.47
7.46
7.45
7.44
7.25
7.06
7.06
7.04
7.04
7.02
7.02
5.59
O
F
N–N
H2N
O
O
CF3
F
IV.b
2.00
2.02
0.62
1.17
0.78
1.03
2.00
f1 (ppm)

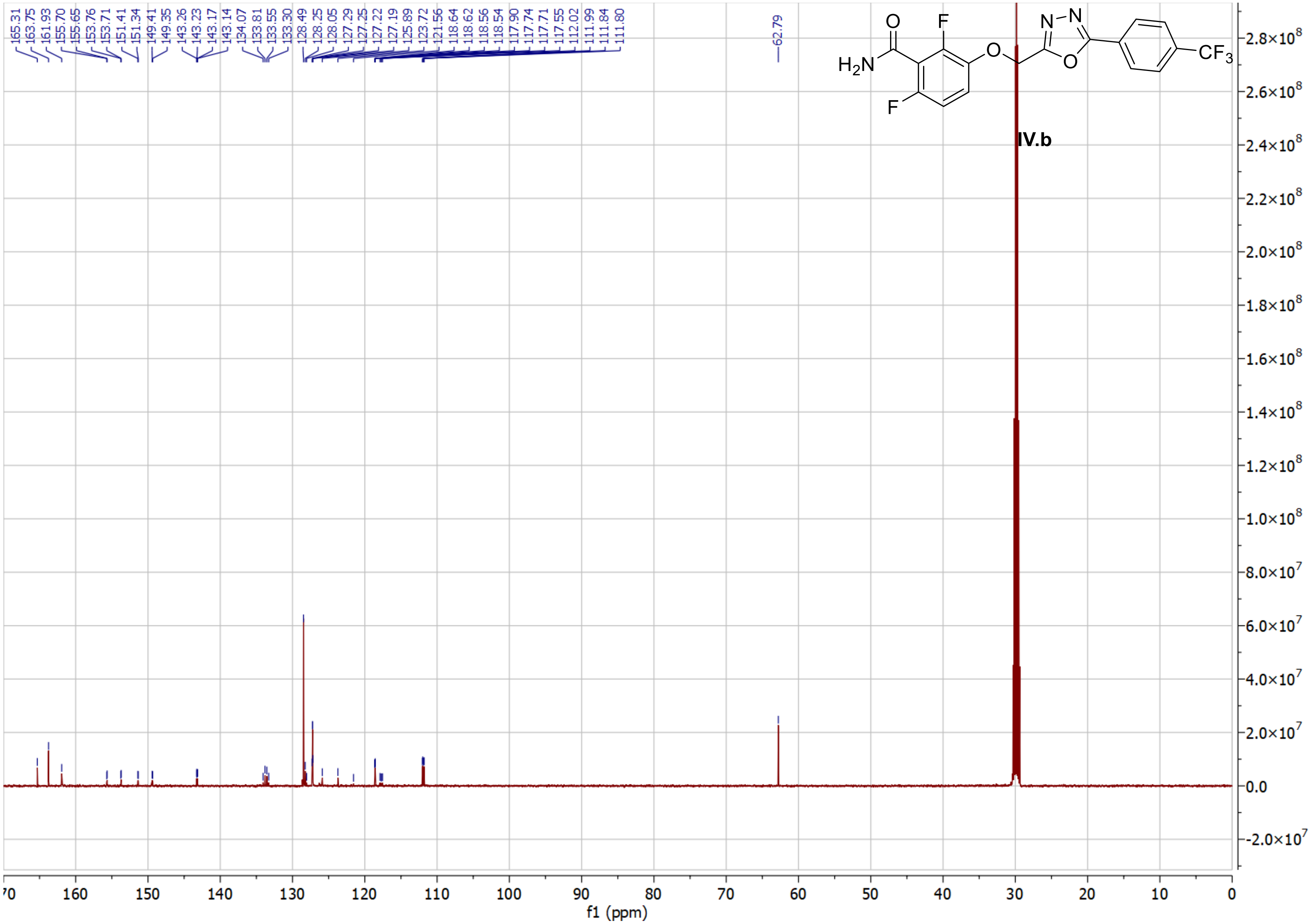
165.31
163.75
161.93
155.70
155.65
153.76
153.71
151.41
151.34
149.41
149.35
143.26
143.23
143.17
143.14
134.07
133.81
133.55
133.30
128.49
128.25
128.05
127.29
127.25
127.22
127.19
125.89
123.72
121.56
118.64
118.62
118.56
118.54
117.90
117.74
117.71
117.55
112.02
111.99
111.84
111.80
62.79
O
F
H_2N
F
O
N
N
O
CF_3
IV.b
f1 (ppm)

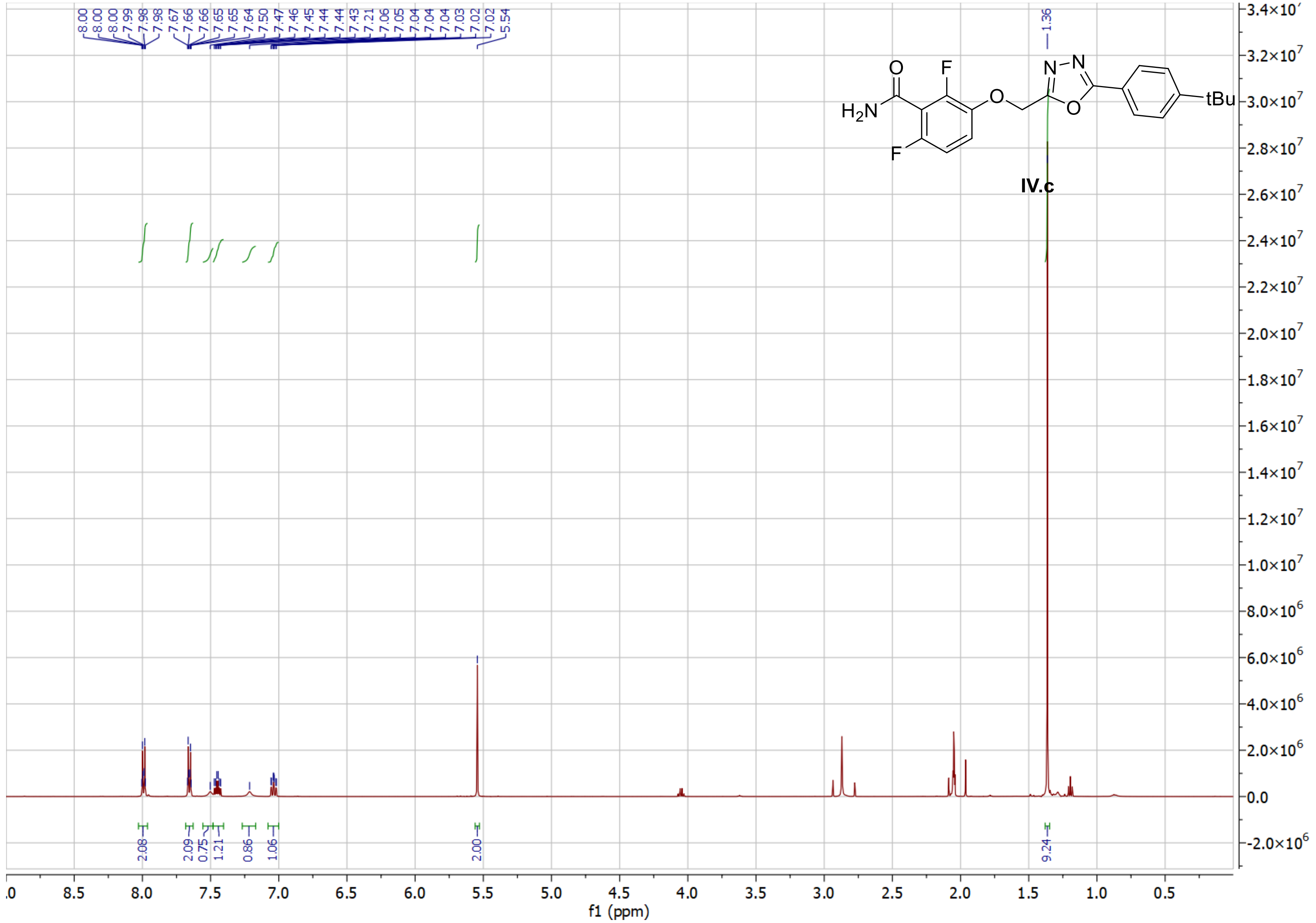

8.00
8.00
8.00
7.99
7.98
7.98
7.67
7.66
7.66
7.65
7.65
7.64
7.50
7.47
7.46
7.45
7.44
7.44
7.43
7.21
7.06
7.05
7.04
7.04
7.04
7.03
7.02
7.02
5.54
1.36
IV.c
H2N
O
F
F
O
N
N
O
tBu
2.08
2.09
0.75
1.21
0.86
1.06
2.00
9.24
f1 (ppm)

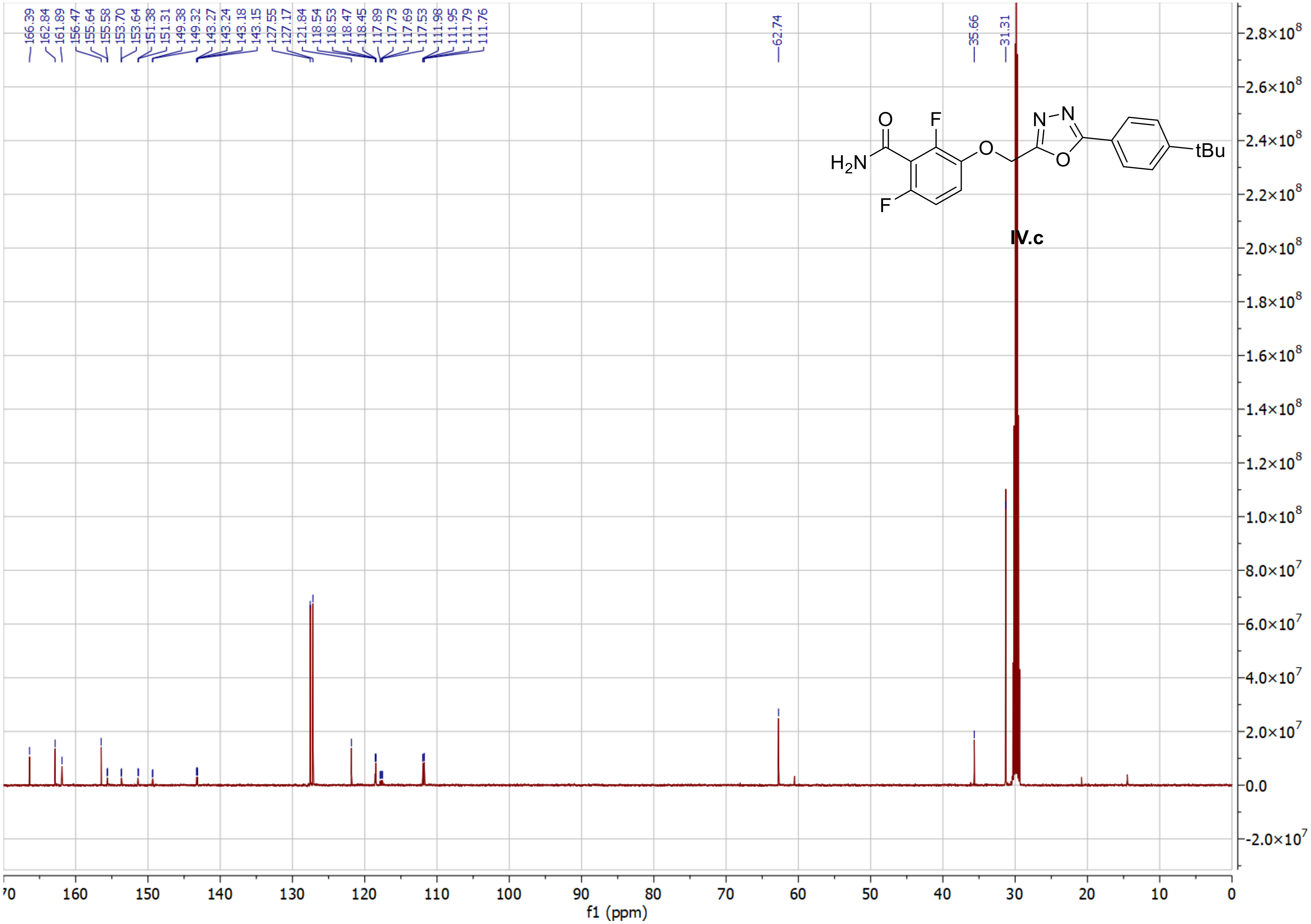
166.39
162.84
161.89
156.47
155.64
155.58
153.70
153.64
151.38
151.31
149.38
149.32
143.27
143.24
143.18
143.15
127.55
127.17
121.84
118.54
118.53
118.47
118.45
117.89
117.73
117.69
117.53
111.98
111.95
111.79
111.76
62.74
35.66
31.31
O
F
N
N
O
O
H_2N
F
tBu
IV.c
2.8×10^8
2.6×10^8
2.4×10^8
2.2×10^8
2.0×10^8
1.8×10^8
1.6×10^8
1.4×10^8
1.2×10^8
1.0×10^8
8.0×10^7
6.0×10^7
4.0×10^7
2.0×10^7
0.0
-2.0×10^7
160
150
140
130
120
110
100
90
80
70
60
50
40
30
20
10
0
f1 (ppm)

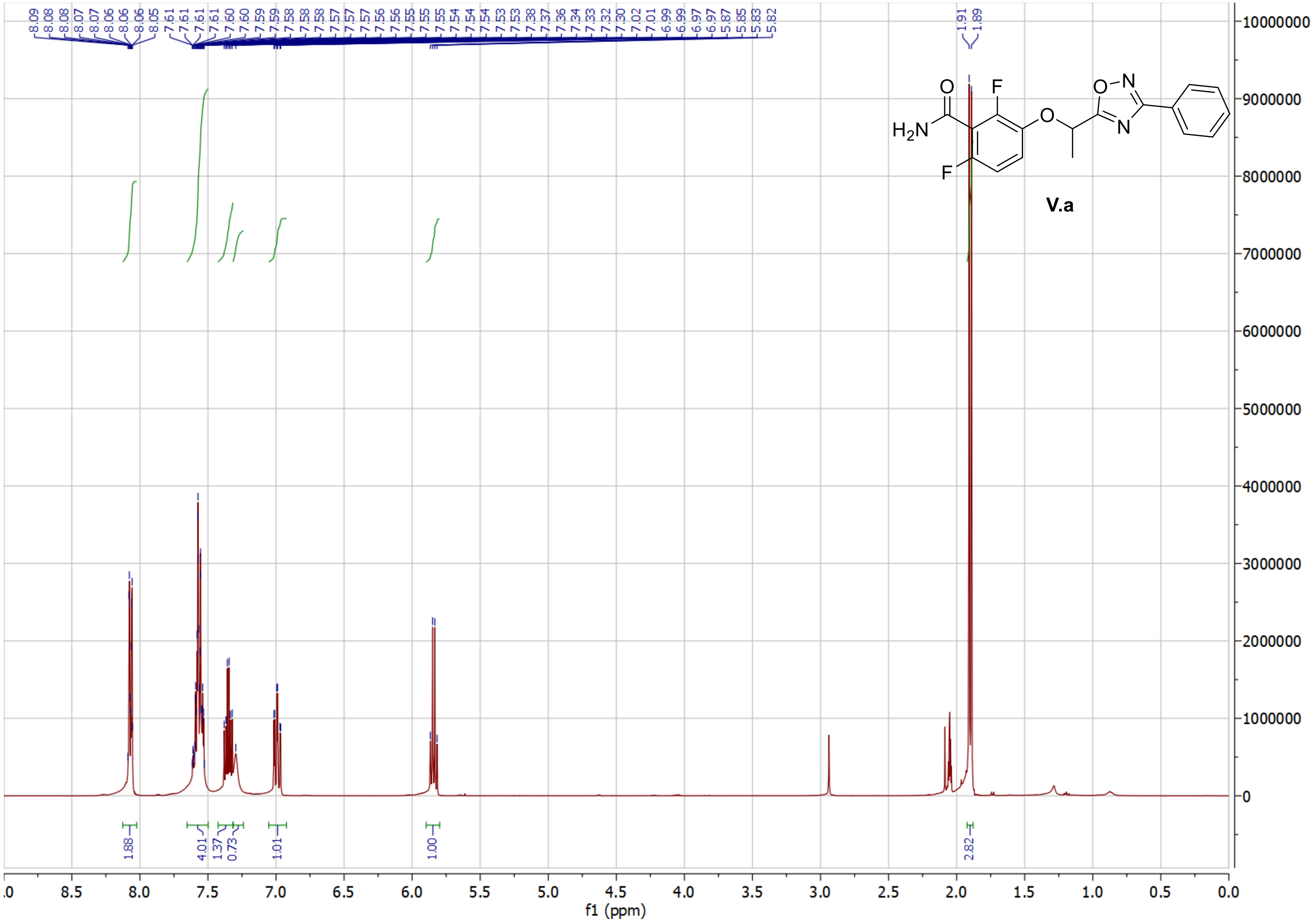
8.09 8.08 8.08 8.07 8.07 8.06 8.06 8.06 8.05 7.61 7.61 7.61 7.61 7.60 7.60 7.59 7.59 7.58 7.58 7.58 7.57 7.57 7.57 7.56 7.56 7.55 7.55 7.55 7.54 7.54 7.54 7.53 7.53 7.38 7.37 7.36 7.34 7.33 7.32 7.30 7.02 7.01 6.99 6.97 6.97 5.87 5.85 5.83 5.82
1.91 1.89
V.a
1.88
4.01
1.37
0.73
1.01
1.00
2.82
f1 (ppm)

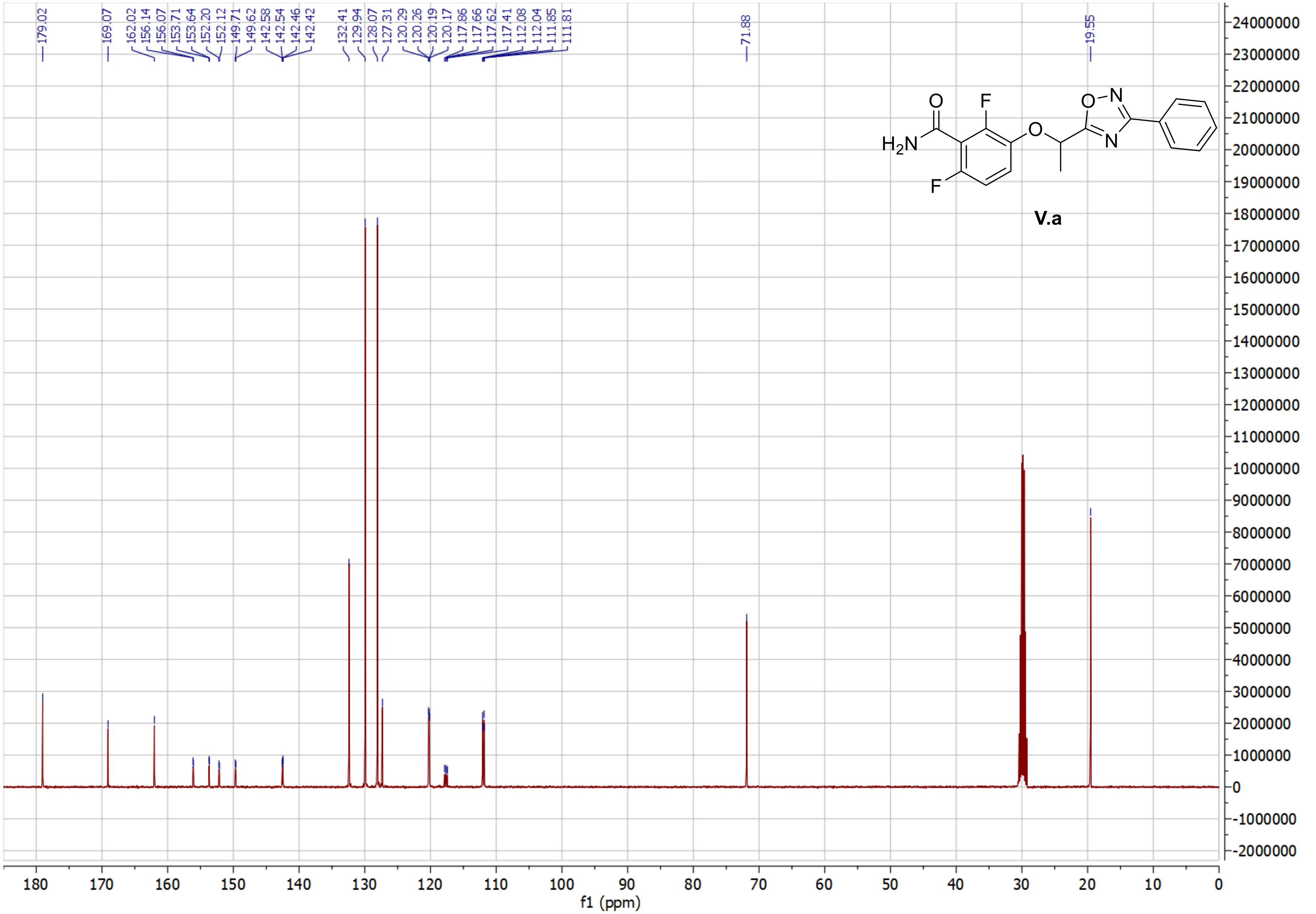
179.02
169.07
162.02
156.14
156.07
153.71
153.64
152.20
152.12
149.71
149.62
142.58
142.54
142.46
142.42
132.41
129.94
128.07
127.31
120.29
120.26
120.19
120.17
117.86
117.66
117.62
117.41
112.08
112.04
111.85
111.81
71.88
19.55
V.a
f1 (ppm)

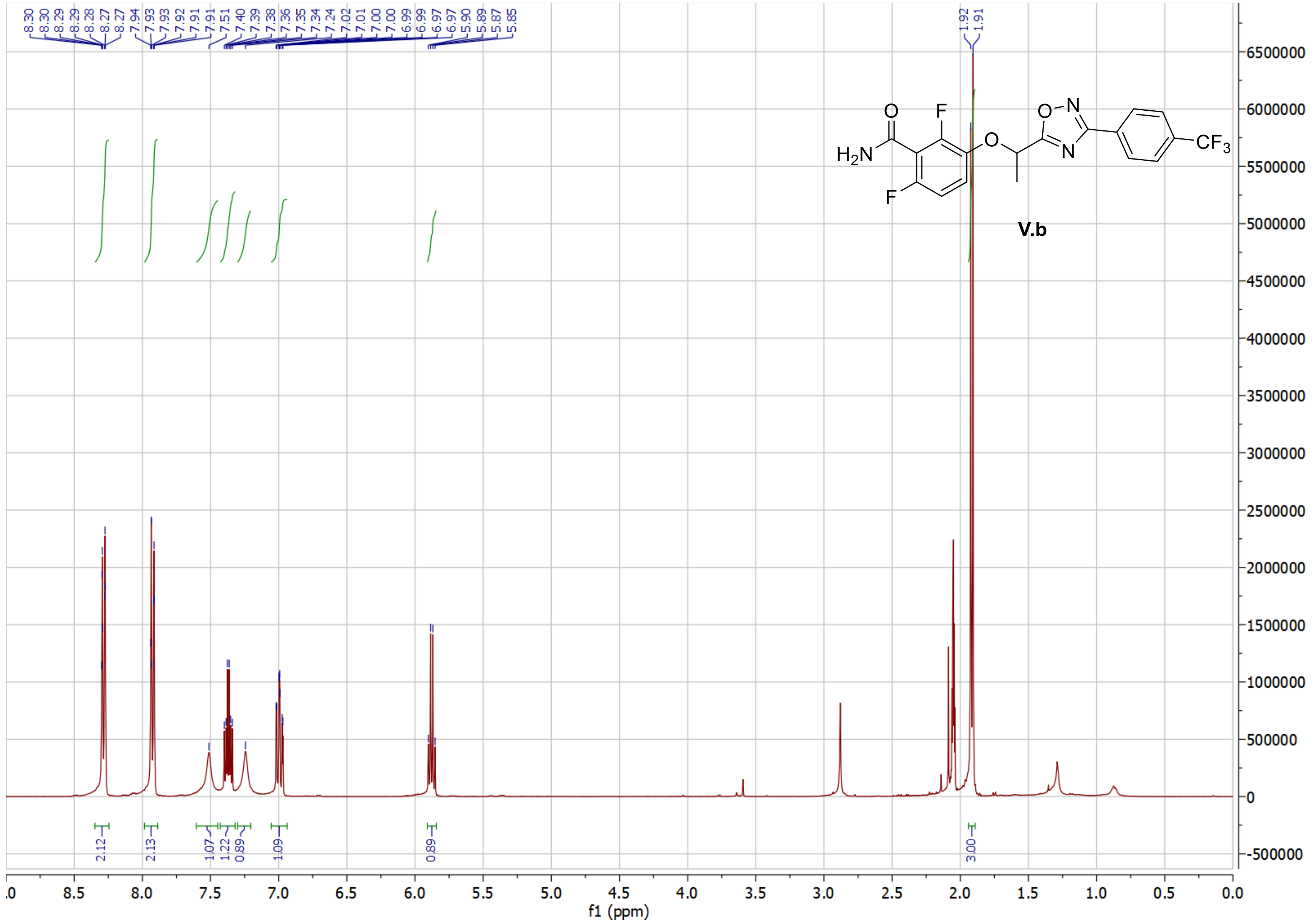

V.b
f1 (ppm)

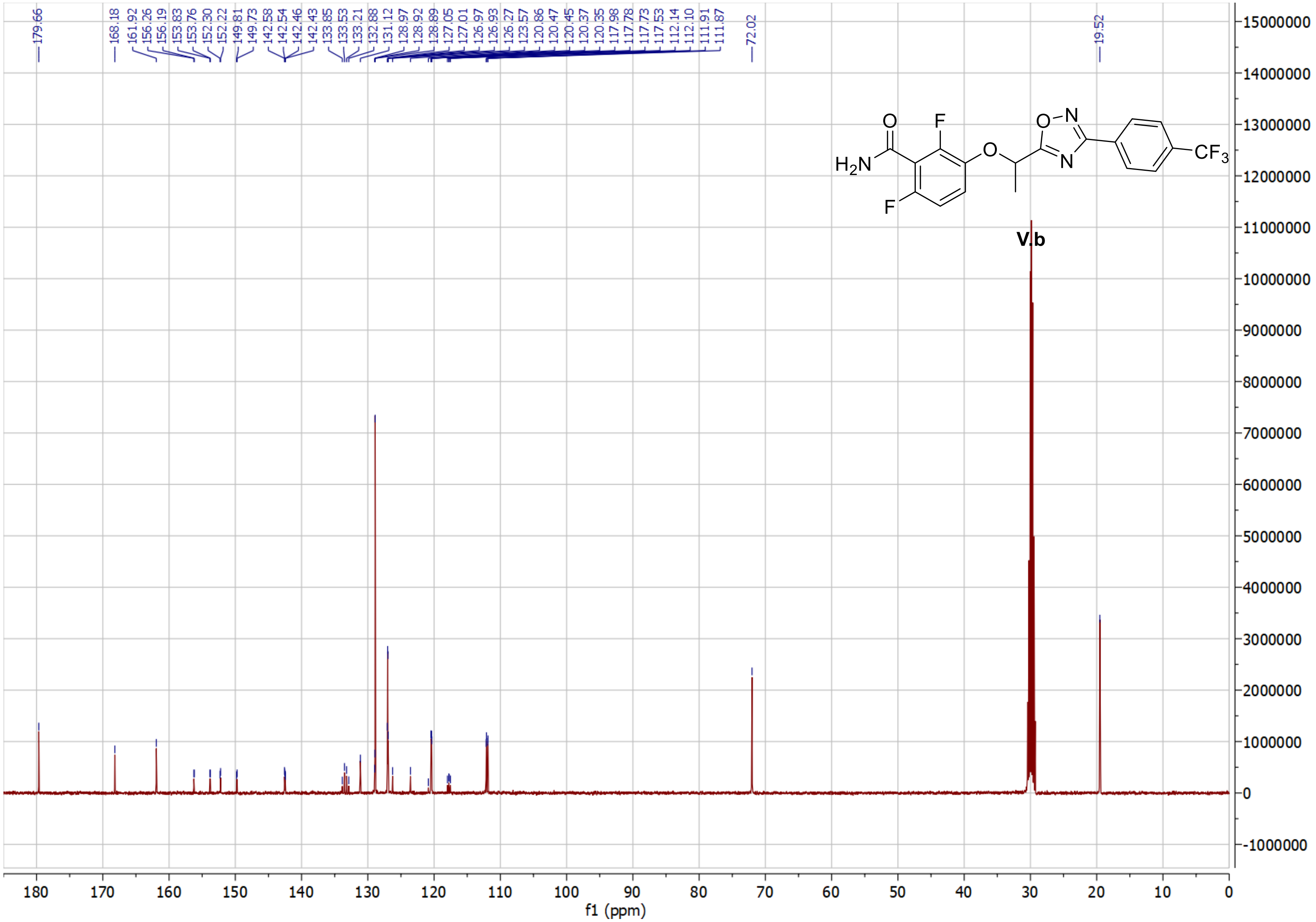

179.66
168.18
161.92
156.26
156.19
153.83
153.76
152.30
152.22
149.81
149.73
142.58
142.54
142.46
142.43
133.85
133.53
133.21
132.88
131.12
128.97
128.92
128.89
127.05
127.01
126.97
126.93
126.27
123.57
120.86
120.47
120.45
120.37
120.35
117.98
117.78
117.73
117.53
112.14
112.10
111.91
111.87
72.02
19.52
O
F
H2N
N
O
O
N
N
CF3
F
f1 (ppm)

V.b

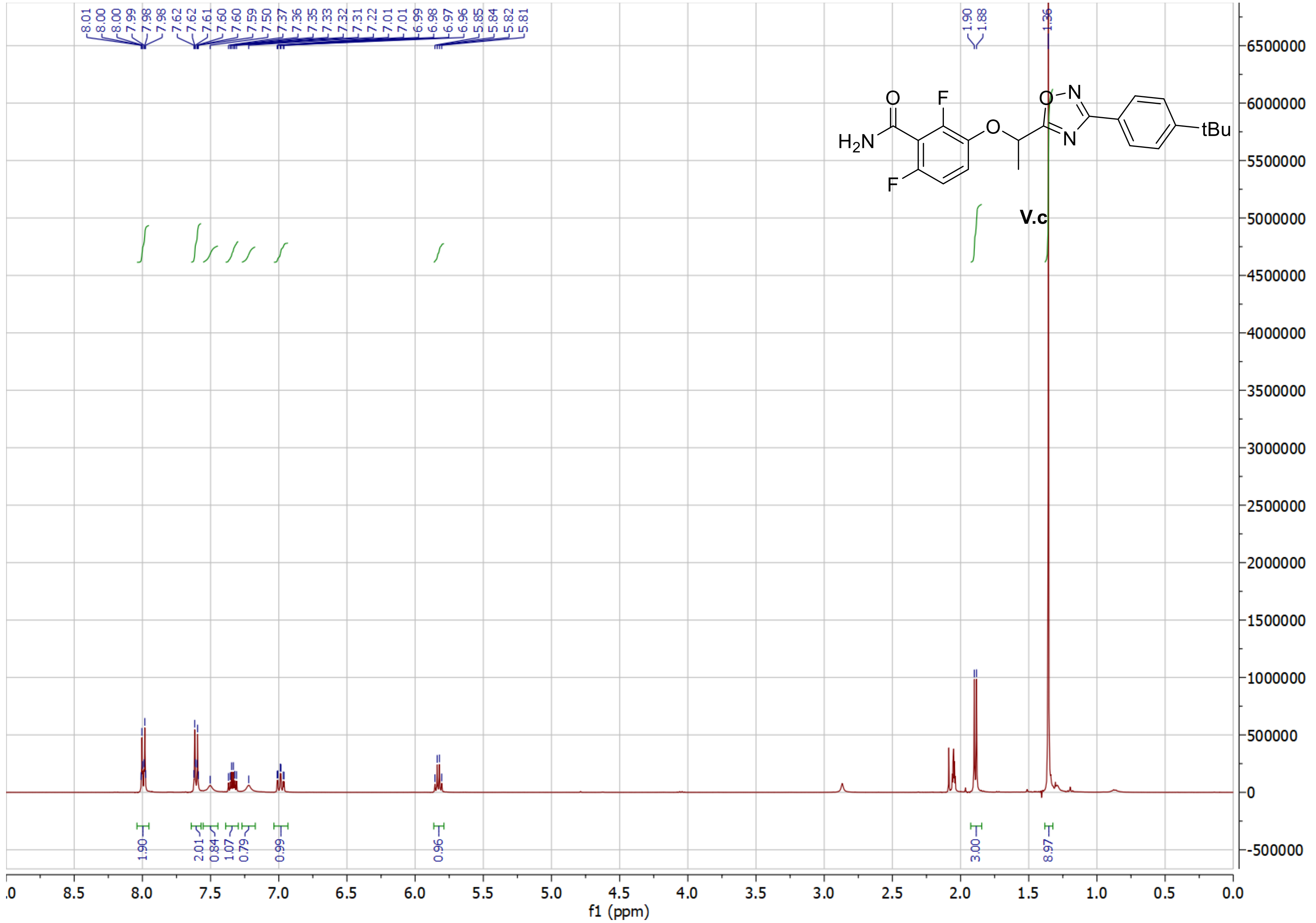

8.01 8.00 8.00 7.99 7.98 7.98 7.62 7.62 7.61 7.60 7.60 7.59 7.50 7.37 7.36 7.35 7.33 7.32 7.31 7.22 7.01 7.01 6.99 6.98 6.97 6.96 5.85 5.84 5.82 5.81
1.90 1.88
1.36
V.c
H2N O F F O O N N tBu
1.90 2.01 0.84 1.07 0.79 0.99 0.96 3.00 8.97
f1 (ppm)

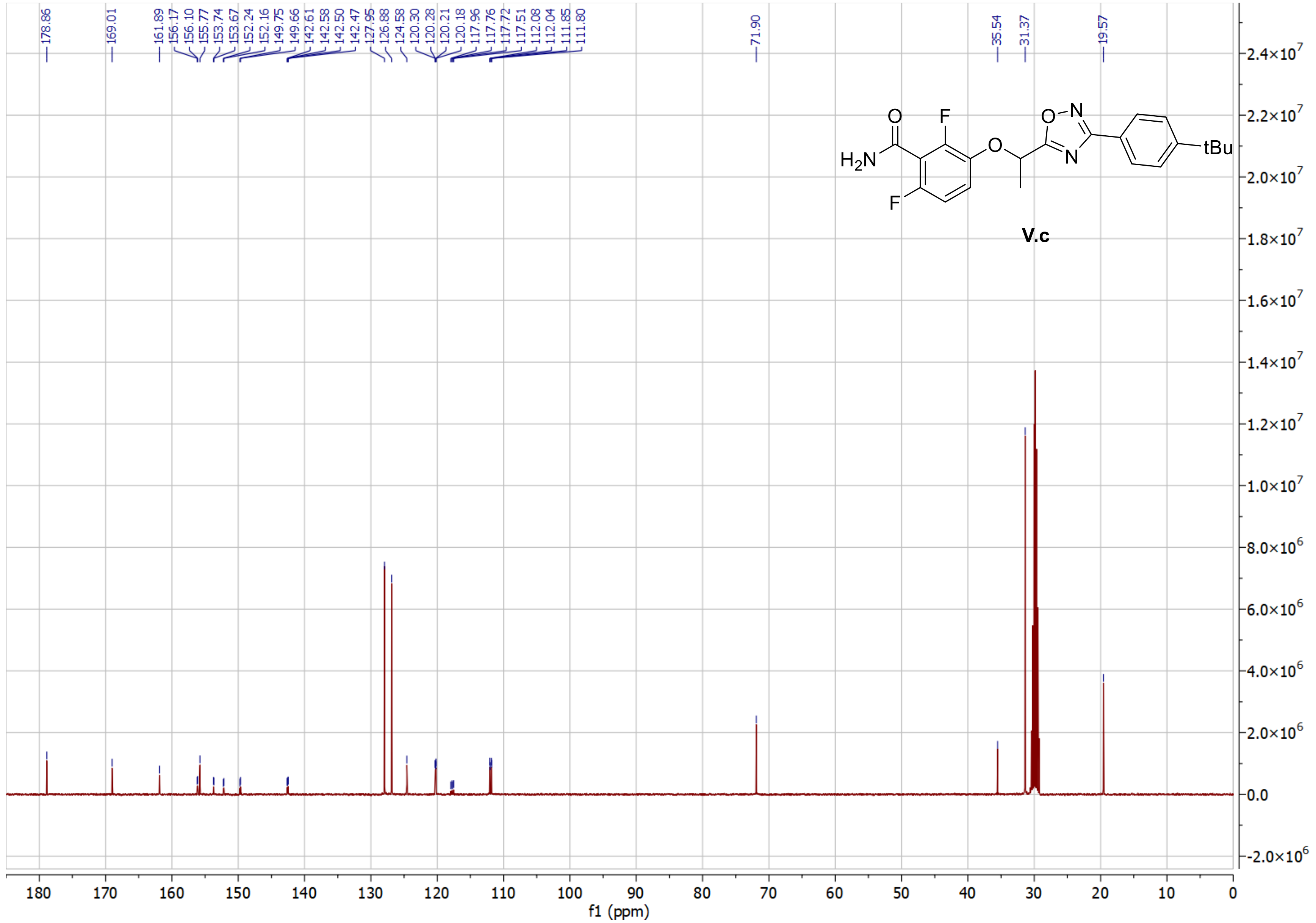
178.86
169.01
161.89
156.17
156.10
155.77
153.74
153.67
152.24
152.16
149.75
149.66
142.61
142.58
142.50
142.47
127.95
126.88
124.58
120.30
120.28
120.21
120.18
117.96
117.76
117.72
117.51
112.08
112.04
111.85
111.80
71.90
35.54
31.37
19.57
O
F
H2N
F
O
O
N
N
tBu
V.c
f1 (ppm)
180
170
160
150
140
130
120
110
100
90
80
70
60
50
40
30
20
10
0
2.4×10^7
2.2×10^7
2.0×10^7
1.8×10^7
1.6×10^7
1.4×10^7
1.2×10^7
1.0×10^7
8.0×10^6
6.0×10^6
4.0×10^6
2.0×10^6
0.0
-2.0×10^6
